# Ultra-processed foods sourced 7-ketositosterol aggravates colitis through gut dysbiosis induced-PDLIM3 activation

**DOI:** 10.1080/19490976.2025.2587980

**Published:** 2025-11-24

**Authors:** Jing Yan, Xiaoqi Pang, Qi Chen, Jingjing Wang, Zimin Wang, Kailin Jiao, Yujie Dai, Ting Xia, Ge Jin, Weilong Zhong, Nan Wang, Bangmao Wang, Jin Zheng, Xin Xu, Hailong Cao

**Affiliations:** a Department of Gastroenterology and Hepatology, Tianjin Medical University General Hospital, National Key Clinical Specialty, Tianjin Institute of Digestive Diseases, Tianjin Key Laboratory of Digestive Diseases, Tianjin, China; b Department of Nutrition, Tangdu Hospital, Fourth Military Medical University, Xi’an, China; c Department of Nutrition, the Third Central Hospital of Tianjin, Tianjin, China; d Department of Nutrition, the First Affiliated Hospital, Air Force Medical University, Xi’an, China; e State Key Laboratory of Food Nutrition and Safety, Key Laboratory of Industrial Fermentation Microbiology, College of Biotechnology, Tianjin University of Science and Technology, Tianjin, China

**Keywords:** Ultra-processed foods, 7-ketositosterol, colitis, gut microbiota, PDLIM3, protein interaction

## Abstract

Excessive ultra-processed foods (UPFs) consumption has been reported to increase the risk of inflammatory bowel disease (IBD). However, the specific mechanisms involved remain unclear. As an important ingredient of UPFs, 7-ketositosterol (KS) is synthesized mainly from high-temperature heated oils. We found that KS intake is higher in IBD patients and is related to disease activity. KS exacerbates colitis in a gut microbiota-dependent manner in mice, altering the gut microbiota composition and increasing the abundance of potential pathogenic bacteria, especially *Staphylococcus lentus* (SL). Moreover, SL aggravates DSS-induced colitis. Mechanically, KS upregulates the expression of PDZ and LIM domain 3 (PDLIM3). SL-derived lysin motif peptidoglycan-binding domain-containing protein (LPDP) interacts with PDLIM3 and activates the p38MAPK/NF-κB signaling pathway. Furthermore, tubuloside B, which is selected by high-throughput screening, blocks the interaction of PDLIM3 and LPDP, and ameliorates SL-aggravated colitis. Our study reveals that KS exposure promotes colitis via the gut microbiota and PDLIM3 interaction, providing evidence of IBD pathogenesis and a potential therapeutic strategy for IBD treatment.

## 1. Introduction

Inflammatory bowel disease (IBD), including ulcerative colitis (UC) and Crohn’s disease (CD), has become a global disease with a high prevalence in Western regions, accelerating its incidence in newly industrialized countries and emergence in developing countries.^1^ It is characterized as a multifactorial-induced, chronic, and relapsing immune-mediated inflammatory disease of the gastrointestinal tract. Environmental factors, altered microbiota, dysregulated immune system, and genetic susceptibility may all play a role in the development of IBD.^1-3^ There is a growing body of clinical and experimental evidence indicates that diet is involved in the pathogenesis of gut inflammation in IBD.^4^^,^^5^ Ultra-processed foods (UPFs) are recognized as energy-dense products with high levels of refined sugar, salt, and fats, but low levels of dietary fiber, vitamins, and minerals. They usually include processed meat, various types of sauce, refined sweetened foods, chocolate, chips, pastries, biscuits, ice cream, and soft drinks. Higher UPFs intake has been identified to be associated with an increased risk of IBD, especially CD, and gut microbiota was regarded as potential mediator of the negative effects of UPFs on metabolism and health.^6-10^ In addition to focusing on high fat, high sugar, or food additives, several studies have shown that some chemicals produced during food processing had toxicity or could induce intestinal inflammation, such as heterocyclic aromatic amines, trans-fatty acid, acrylamide, and advanced glycation end products.^11-14^

Phytosterols naturally exist in plants, such as cereals, nuts, legumes, vegetables, fruits, and tubers, and especially abundant in seed oils.^15^ They possess protective effects on health, thus have been supplemented in functional food products.^16-18^ Nonetheless, phytosterols are prone to being autoxidized under conditions as heat, light, storage, and food processing, especially high-temperature cooking, which leads to higher amounts of phytosterol oxidation products (POPs) in fried and baked foods such as French fries, potato crisps, hamburgers, spreads, cakes, egg rolls and cookies.^19^^,^^20^ There are many kinds of phytosterols, mainly including sitosterol, campesterol, stigmasterol, and brassicasterol. Sitosterol is the predominant phytosterol.^21^ Oxidized forms of phytosterols, primarily consisting of hydroxyl, ketone, and epoxy phytosterols, increase the synthesis of various POPs in vivo.^15^

Previous studies have indicated that POPs exposure may have adverse effects on cell and animal models. 7-Keto-stigmasterol was reported to increase the production of TNF-*α* and IL-8 in Caco-2 cells.^22^ 7β-Hydroxy-sitosterol/campesterol and 7-keto-sitosterol/campesterol were found to promote TNF-*α* secretion in bone marrow-derived macrophages in another study.^23^ POPs were also shown to increase the ROS production in vitro and in vivo studies.^24^^,^^25^ However, it remains unknown whether POPs could promote gut dysbiosis and exacerbate colon inflammation. 7-Ketositosterol (KS) was shown to account for the greatest proportion of the mixture of POPs.^24^^,^^26^ Therefore, we aimed to explore the effects of KS on colitis and potential mechanisms.

We hypothesize that exposure to POPs could exacerbate colitis and modulate the gut microbiota. To verify this, we surveyed the intake of fried and baked food among patients with IBD prior to the onset of the disease. KS, synthesized from *β*-sitosterol, is given to mice to evaluate the effect on colitis. Our study revealed that patients with IBD tend to consume more fried and baked foods, leading to higher exposure to KS. KS has been shown to worsen colitis and increase the expression of PDZ and LIM 3 domain (PDLIM3), which depends on the gut microbiota. This increase in PDLIM3 is associated with increased levels of *Staphylococcus lentus*, which possesses a protein called Lysin motif (LysM) peptidoglycan-binding domain-containing protein (LPDP) that interacts with PDLIM3 to activate the p38MAPK/NF-κB (p38 mitogen-activated protein kinase/nuclear factor kappa-light-chain enhancer of activated B cells) pathway, ultimately exacerbating inflammation. Our study provides new insight into the underlying mechanisms between KS and gut microbiota, and suggests that PLDIM3 could be a potential intervention for the treatment of KS-aggravated colitis.

## 2. Results

### 2.1 Patients with IBD consume more ultra-processed foods containing KS

**Figure 1.**
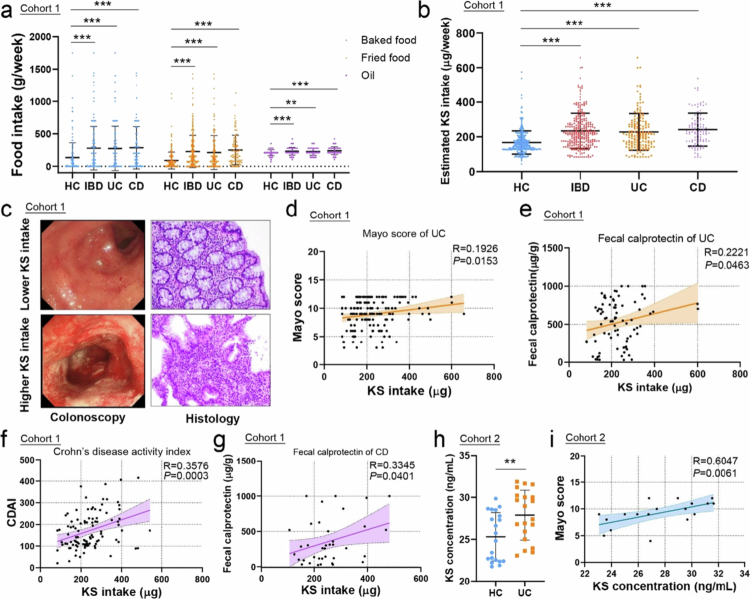
KS intake is higher in patients with IBD and is associated with disease activity. (a) Comparison of the dietary intake of baked food, fried food, and oil in Healthy Control (HC) group (*n* = 296), UC patients (*n* = 158), and CD patients (*n* = 100). (b) Estimated KS intake in the HC group (*n* = 296), total IBD patients (*n* = 258), UC patients (*n* = 158), and CD patients (*n* = 100) based on dietary intake, cohort 1. (c) Endoscopic images and histopathological pictures from UC patients with relatively high or low KS exposure. (d, e) Correlation between KS intake and Mayo score (*n* = 158) and fecal calprotectin (*n* = 81) in UC patients. (f, g) Correlation between KS intake and disease activity index (*n* = 96) and fecal calprotectin in CD patients (*n* = 38). (h) KS concentration in serum samples from healthy controls (*n* = 20) and patients with new-onset UC (*n* = 20). (i) Correlation between the KS concentration and disease activity of UC patients in cohort 2. KS 7-ketositosterol. All the data are presented as the mean ± SEM. **P < 0.01; ***P < 0.001. Two-tailed Mann‒Whitney U test in a‒b; Spearman correlation analysis in d‒g and i.

In cohort 1, a total of 554 individuals were recruited, including 296 healthy controls (HC), 158 UC patients and 100 CD patients. The patients we included were primarily inpatients. The basic characteristics and dietary information are shown in Supplementary Table 1. The significant differences in age, gender, smoking, physical activity, and diet were observed between patients with IBD before illness and HC reflected the complexity of IBD occurrence. Additionally, UC patients had a slightly higher body mass index (BMI) compared to HC. However, there was no significant difference in BMI between CD patients and HC. IBD patients consumed more refined grains but fewer coarse grains and vegetables than healthy individuals. We also found that many UC patients could not tolerate dairy products, especially milk, before their illness (Supplementary Table 1). Obviously, the intake of fried and baked food in IBD patients was much higher than that in healthy individuals prior to the onset of their illness (Figure 1a), which might result in increased oil and energy intake in IBD patients. After KS intake was calculated, patients with UC and CD both showed higher KS intake than healthy controls (Figure 1b). Patients with higher KS intake had more severe inflammation according to colonoscopy and histology (Figure 1c, Figure S1a). Additionally, KS intake was positively correlated with disease severity and the level of calprotectin in patients with both UC and CD (Figure 1d–g). In cohort 2, the KS concentration was greater in UC patients than in healthy individuals, and was also positively correlated with the Mayo score (Figure 1h–i).

### 2.2 KS exacerbates colitis and impairs intestinal barrier in mice

**Figure 2.**
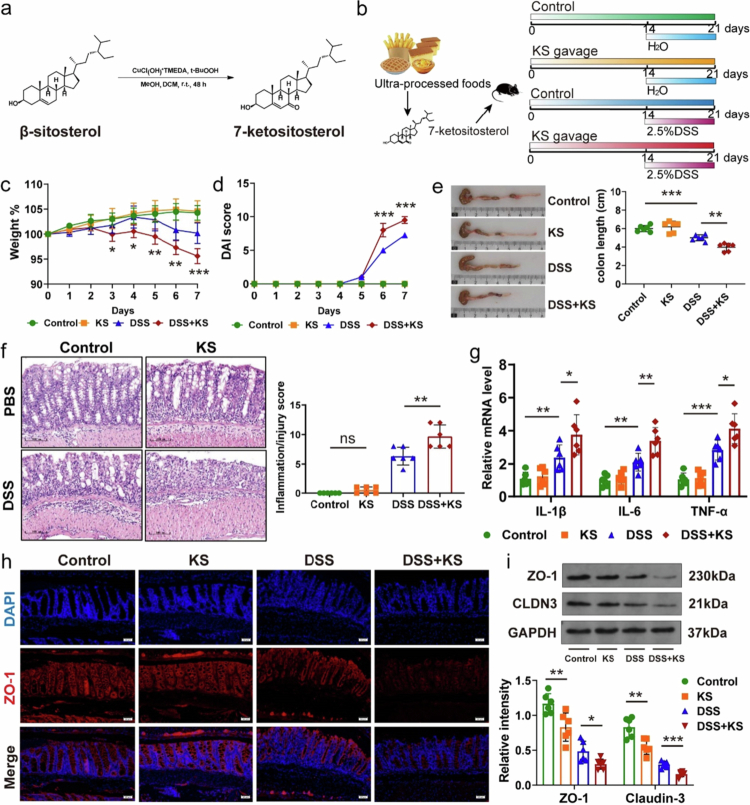
KS exposure aggravates DSS-induced colitis in mice. (a) Synthesis of KS from *β*-sitosterol. (b) The experimental design of DSS-induced colitis model. KS was administered by gavage (100 mg/kg/d, 200 μl/mouse) for 2 weeks before the induction of colitis with 2.5% DSS for 7 d. The mice were continuously exposed to KS during DSS. (c, d) Body weight curves (c) and DAI scores (d) after DSS induction (*n* = 6). (e) Colon length (cm) on day 8 after DSS induction. (f) Representative images of hematoxylin and eosin (H&E)-stained colon sections (left panel) and colon histopathological scores (right panel). Scale bar, 100 μm. (g) Relative mRNA levels of IL-1β, IL-6, and TNF-*α* in the colon. (h) Immunofluorescence staining for ZO-1 (red) and nuclei by DAPI (blue) in the colon. Scale bar, 50 μm. (i) Protein levels of ZO-1 and CLDN3 were measured by Western blotting (*n* = 6). KS 7-ketositosterol, DSS dextran sodium sulfate, ZO-1 zonula occludens-1, CLDN3 Cluaudin 3. Data are presented as the mean ± SEM; *P < 0.05, **P < 0.01, ***P < 0.001 by two-tailed one-way ANOVA.

To investigate the effect of KS on colitis, we synthesized KS from *β*-sitosterol (Figure 2a). KS was given to the mice by gavage, and then 2.5% DSS was administered (Figure 2b). There was no substantial difference in diet, water consumption, and weight between the KS group and the control group. Mice in the DSS + KS group lost more weight compared to the DSS group. And the mice in the DSS + KS group showed shorter colon length and increased disease score. There was no noticeable difference in weight loss, colon length, or disease activity index (DAI) score between the control group and the KS group. Hematoxylin and eosin (H&E) staining showed decreased depth of crypts and more inflammatory cell infiltration in the DSS + KS group compared with DSS group (Figure 2c-f). Meanwhile, the expression of inflammatory cytokines such as IL-1β, IL-6 and TNF-*α* was increased in the colons of DSS + KS mice (Figure 2g, Figure S2a).

The number of goblet cells, as indicated by PAS staining, and mucin production, indicated by mucin-2-positive cells, were subsequently examined. These components represented the main components of the intestinal mucus barrier. We observed a decrease in goblet cells and MUC-2 positive cells in the DSS + KS group compared to the DSS group (Figure S2b and c). Additionally, the effects of KS intake on colon tight junction proteins was assessed, including zonula occludens-1 (ZO-1) and claudin-3 (CLDN3), which are key proteins that maintain gut barrier integrity. The protein expression levels of ZO-1 and CLDN-3 exhibited a significant decrease in the DSS + KS group (Figure 2i). The loss of ZO-1 in the colon was further demonstrated through immunofluorescence staining (Figure 2h). Taken together, these findings indicated that KS exacerbated DSS-induced colitis and disrupted intestinal barrier integrity. KS also aggravated TNBS-induced colitis (Figure S2d–g).

### 2.3 KS alters the composition and diversity of the gut microbiota in mice

**Figure 3.**
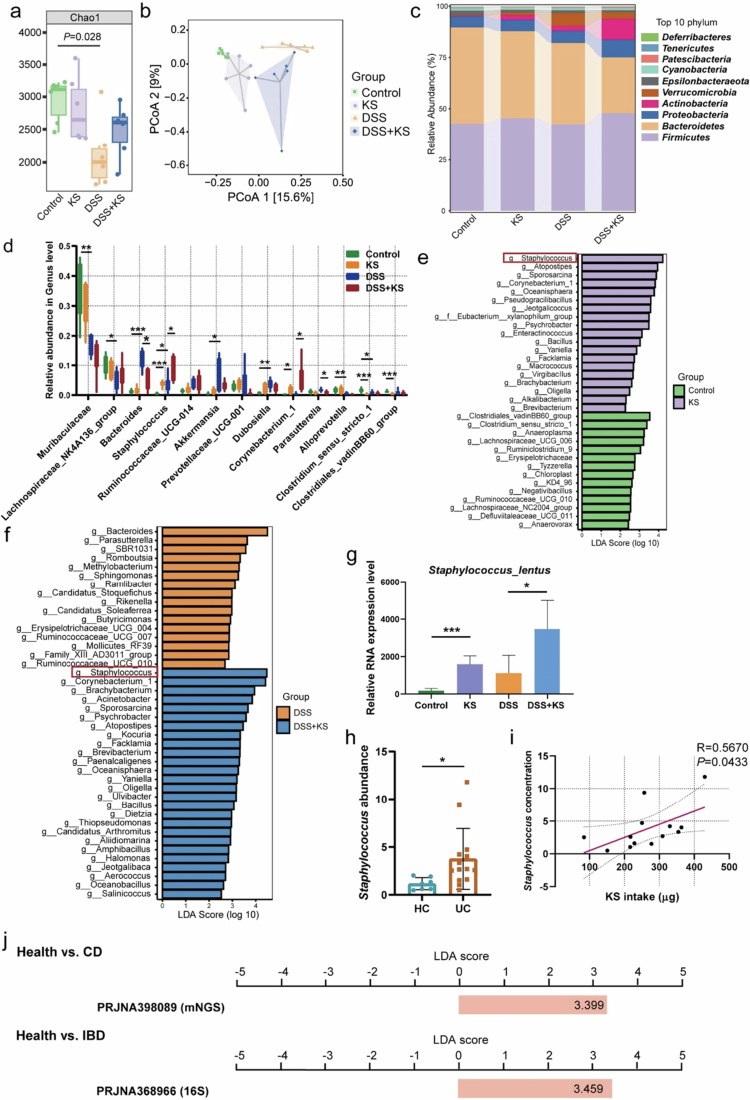
KS alters the gut microbiota composition in DSS-induced mice. The mice were treated as described in Figure 2b, and total fecal bacteria were detected via 16S rRNA sequencing. (a) *α* diversity was measured via the Chao 1 diversity index. (b) Principal coordinate analysis (PCoA) based on the unweighted UniFrac distance. (c) Relative abundance of microbial taxa at the phylum level. (d) Relative abundance of microbial taxa at the genus level. (e) Histogram of the LDA scores of significantly different bacterial taxa at the genus level between the control and KS groups (LDA score ≥ 2). (f) Histogram of the LDA scores of significantly different bacterial taxa at the genus level between the DSS and DSS + KS groups. (g) Relative RNA level of *Staphylococcus lentus* (*n* = 6) in each group. (h) The abundance of *Staphylococcus* in healthy individuals (*n* = 7) and UC patients (*n* = 14). (i) The correlation between *Staphylococcus* concentration and KS intake (*n* = 13). (j) The abundance of *Staphylococcus* in the gut microbiota of CD and IBD patients compared to healthy controls based on GMrepo data. Data are presented as the mean ± SEM; *P < 0.05, **P < 0.01, ***P < 0.001 by two-tailed one-way ANOVA.

To investigate the alteration of gut microbiota after KS administration, fresh feces from the four groups were collected for 16S rRNA sequencing. The gut microbiota showed similarities and differences among the four groups (Figure S3a). The Chao1 index, an index of *α* diversity indicating microbiota richness, showed differences between the control and DSS group (*P* = 0.028), but there were no dramatic differences between the DSS group and the DSS + KS group (Figure 3a). The *β* diversity was characterized via principal coordinate analysis (PCoA) and unweighted UniFrac distances, which reflected the differences in the composition of the microbiota among the groups. PCoA revealed that there were differences among the four groups (Figure 3b). At the phylum level, the proportion of *Actinobacteria* (*P* = 0.023 for KS group, *P* = 0.002 for DSS group) and *Verrucomicrobia* (*P* = 0.075 for KS group, *P* = 0.024 for DSS group) phyla was increased in the KS and DSS groups compared with the control group, while the proportion of *Bacteroidetes* phylum was decreased in the KS and DSS groups (*P* = 0.407 for KS group, *P* = 0.095 for DSS group). Meanwhile, the ratio of *Firmicutes* to *Bacteroidetes* was increased in the KS and DSS group with no statistical difference (*P* = 0.424 for KS group, *P* = 0.488 for DSS group). In contrast, the proportion of *Actinobacteria* phylum was greater in the DSS + KS group compared with the DSS group (*P* = 0.020). However, the proportion of the *Bacteroidetes* phylum was substantially decreased in the DSS + KS group (*P* = 0.004). And the ratio of *Firmicutes* to *Bacteroidetes* was significantly increased in the DSS + KS group (*P* = 0.009) (Figure 3c).

The genus-level analysis revealed that the potential pathogenic bacteria *Staphylococcus*, *Corynebacterium_1*, *Pseudogracilibacillus*, *Psychrobacter*, *Sporosarcina*, *Atopostipes* were remarkably enriched, while beneficial bacteria such as *Ruminococcaceae_UCG-010*, short-chain fatty acids-producing bacteria, *Clostridium_sensu_stricto_1*, *Clostridiales_vadinBB60_group* and *Lachnospiraceae_UCG-006* were decreased in the KS group compared to the control group. In addition, *the abundances of Bacteroides*, *Staphylococcus*, *Ruminococcaceae_UCG_014*, *Akkermansia* and *Dubosiella* were substantially greater in the DSS group compared to control group. Furthermore, in the DSS + KS group, *Staphylococcus* and *Corynebacterium_1* were remarkably enriched compared to the DSS group, especially *Staphylococcus*, while *Bacteroides* and *Parasutterella* were decreased. Notably, *Muribaculaceae* generally tapered off in the KS, DSS and DSS + KS groups compared to those in the control group. *Staphylococcus* and *Corynebacterium_1* were significantly increased in the KS and DSS + KS groups, suggesting that potential pathogenic bacteria might be a driving force for the induction of inflammation (Figure 3d–f, Figure S3b, c). Further screening at the species level revealed that the changes in *Staphylococcus* contributed mainly to *Staphylococcus lentus*. We found that the abundance of *Staphylococcus lentus* was significantly greater in mice of the KS and DSS + KS groups compared with that of the control and DSS groups, respectively (Figure 3g). We subsequently tested the abundance of *Staphylococcus* in healthy individuals and UC patients and found that the abundance of *Staphylococcus* was higher in UC patients than in healthy controls (Figure 3h). Further analysis demonstrated that KS intake was positively correlated with the abundance of *Staphylococcus* (Figure 3i). Consistent with our findings, analysis of data from the Gmrepo database showed that *Staphylococcus* is a marker taxon with higher abundance in CD and IBD patients than in healthy control (Figure 3j). However, KS did not directly promote the growth of *Staphylococcus lentus in vitro* (Figure S3d).

In addition, the correlation between the intestinal microbiota and intestinal inflammation was analyzed by comparing the colon length, DAI score, inflammatory score, and inflammatory cytokines. Our findings showed that certain bacteria such as *Staphylococcus*, *Akkermansia*, *Corynebacterium_1*, and *Faecalibaculum* were positively correlated with intestinal inflammation. On the other hand, bacteria such as *Muricaculaceae*, *Prevotellaceae_UCG−001*, and *Alloprevotella* were negatively correlated with inflammation (Figure S3e).

### 2.4 KS increases the expression of PDLIM3 and activates the p38MAPK/NF-κB pathway to aggravate colitis

**Figure 4.**
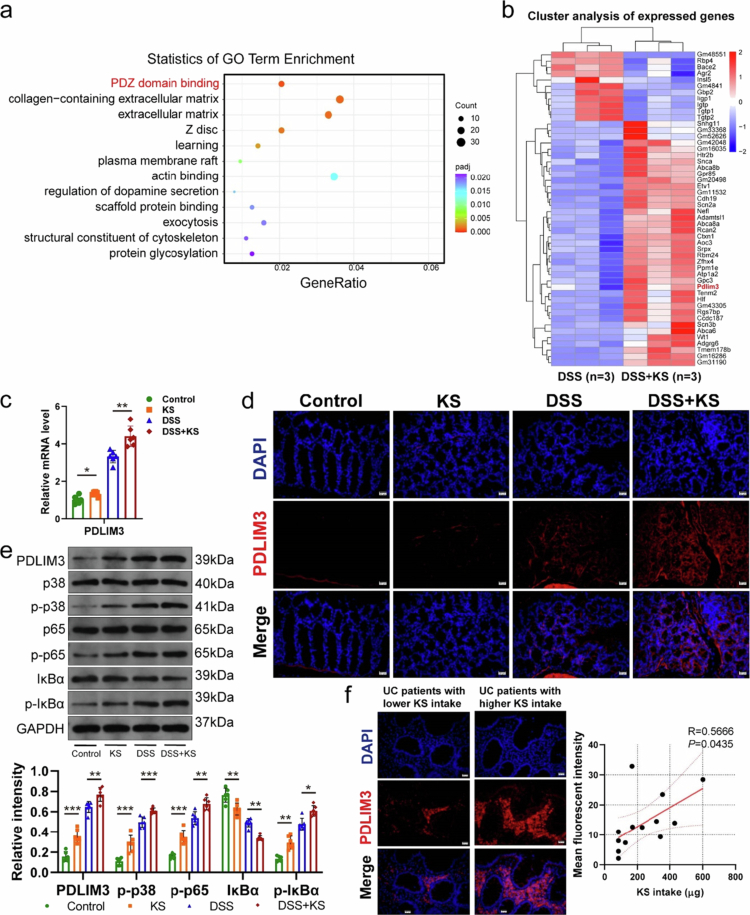
KS upregulates the expression of PDLIM3 and activates the p38MAPK/NF-κB pathway. (a) GO enrichment analysis of genes significantly upregulated in colon tissues. (*n* = 3). (b) Heatmap representation of RNA sequencing data from colon tissues (*n* = 3). (c) Relative mRNA level of PDLIM3 (*n* = 6). (d) Immunofluorescence staining for PDLIM3 (red) and nuclei by DAPI (blue) in the colon. Scale bar, 20 μm. (e) Protein levels of PDLIM3, p38, *p*-p38, p65, *p*-p65, IκBα, and *p*-IκBα were measured via Western blotting (*n* = 6), and GAPDH was used as a loading control. (f) Immunofluorescence staining for PDLIM3 (red) and nuclei by DAPI (blue) in the colon of UC patients with low or high KS intake and correlation analysis between KS intake and fluorescence intensity (*n* = 13). GO gene ontology, PDLIM3 PDZ and LIM domain 3. Data are presented as the mean ± SEM; *P < 0.05, **P < 0.01, ***P < 0.001 by two-tailed one-way ANOVA.

To further identify the substantial changes responsible for the adverse effects of KS on the colon, RNA sequencing of colon tissues from mice in the DSS and DSS + KS groups was conducted. We detected 129 downregulated and 629 upregulated genes in the mice in the DSS + KS group compared with those in the DSS group (Figure S4a and b). Gene ontology (GO) enrichment analysis revealed enrichment in a series of molecular functions, cellular components, and biological processes. Specifically, PDZ domain binding showed significant enrichment (Figure 4a). By combining cluster analysis with differential gene expression and gene expression levels, we found that the expression of PDLIM3 was higher after KS treatment in the DSS model (Figure 4b). PDLIM3 is also a protein localized in Z-discs, which was shown to be enriched in the GO analysis (Figure S4c). PDLIM3, which belongs to a family of proteins, PDLIM1-7, has been reported to play an important role in cytoskeleton and signal transduction.^^27^^,^^28^^ Subsequently, the expression of PDLIM3 was demonstrated by real-time PCR, Western blotting, and immunofluorescent staining, and the results showed that the expression of PDLIM3 in intestinal epithelial cells and the intercellular matrix increased after KS treatment and was higher in DSS + KS group than in the DSS group (Figure 4c-e). Similarly, UC patients with increased KS exposure exhibited higher PDLIM3 expression in colonic epithelial cells (Figure 4f). In our study, the MAPK signaling pathway was markedly upregulated according to enrichment analysis of the KEGG pathway (Figure S4d). Western blotting showed that the protein levels of *p*-p38, *p*-p65, and *p*-IκBα were up-regulated in the KS group and were significantly increased in DSS and DSS + KS groups (Figure 4e). The increasing trends of *p*-p38, *p*-p65, and *p*-IκBα were consistent with those of PDLIM3. We also found that the expression of PDLIM3 from colon of IBD patients was higher than that of healthy controls in the RNA sequencing dataset, which confirmed the importance of our findings (Figure S5a).

### 2.5 KS aggravates colitis in mice via the gut microbiota

**Figure 5.**
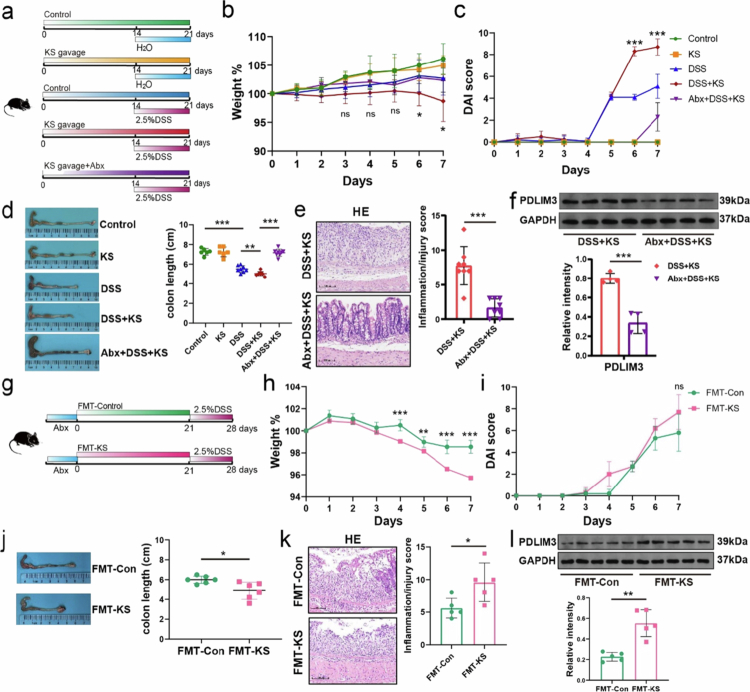
KS aggravates colitis in mice via the gut microbiota. (a) Schematic illustration of the experimental procedure. Abx: antibiotic cocktail. (b, c) Changes in body weight and DAI score after DSS induction (*n* = 6 for the control, KS; *n* = 8 for the DSS, DSS + KS, and Abx + DSS + KS). (d) Colon length (cm) on day 8 after DSS induction. (e) Representative images of hematoxylin and eosin (H&E)-stained colon sections (left panel) and colon histopathological scores (right panel). (*n* = 8) Scale bar, 100 μm. (f) Protein levels of PDLIM3 were measured by Western blotting, and GAPDH was used as a loading control (*n* = 4). (g) Schematic illustration of the FMT procedure. Mouse feces from the KS/Control group were transplanted into recipient mice, and then DSS was administered (*n* = 5). (h, i) Body weight and DAI score. (j) Colon length (cm) on day 8 after DSS induction. (k) Representative images of hematoxylin and eosin (H&E)-stained colon sections (left panel) and colon histopathological scores (right panel). Scale bar, 100 μm. (l) Protein levels of PDLIM3 were measured by Western blotting (*n* = 5). KS 7-ketositosterol, DSS dextran sodium sulfate, FMT fecal microbiota transplantation, PDLIM3 PDZ and LIM domain 3. Data are presented as the mean ± SEM; *P < 0.05, **P < 0.01, ***P < 0.001, ns not significant by two-tailed one-way ANOVA.

To explore the potential influence of gut dysbiosis on the exacerbation of DSS-induced colitis by KS, we added a group of mice treated with an antibiotic cocktail (Figure 5a). The mice were also administered KS and DSS. The weight loss of the antibiotic-treated mice (Abx + DSS + KS group) was lower than that of the DSS + KS group (Figure 5b). No difference was observed in the DAI score or colon length between the Abx + DSS + KS group and the control and KS groups (Figure 5c and d). The weight change in the DSS group was not pronounced compared to colon length and DAI groups, which might be attributed to the influence of food or water intake, as well as defecation status. Similarly, the inflammatory score in antibiotic-treated mice was much lower than that in the DSS + KS group (Figure 5e). The PDLIM3 level of the Abx + DSS + KS group was further detected to explore whether the expression of PDLIM3 was affected by the gut microbiota. A significantly decreased PDLIM3 level was observed in the Abx + DSS + KS group compared with the DSS + KS group (Figure 5f). These findings were also confirmed by immunofluorescent staining, indicating that the upregulation of PDLIM3 might be mediated by the gut microbiota (Figure S6a).

Moreover, FMT experiment was performed to explore whether alterations in the microbiota caused by KS contributed to DSS-induced colitis (Figure 5g). The results exhibited that mice in the FMT-KS group experienced greater weight loss compared to the FMT-Con group (Figure 5h). Additionally, the DAI score was higher and the colon length was shorter in mice of FMT-KS group (Figure 5i and 5j). Histopathological damage also revealed that mice that received fecal microbiota from KS-treated donors exhibited more severe colitis (Figure 5k). The expression of PDLIM3 was higher in mice of FMT-KS group compared to that of FMT-Con group in the FMT experiment, which further demonstrated that the upregulation of PDLIM3 might be mediated by the gut microbiota (Figure 5l, Figure S6b). These results demonstrated that gut dysbiosis mediated by KS exposure rather than KS itself, exacerbated DSS-induced colitis in mice.

### 2.6 *Staphylococcus lentus*exacerbates DSS-induced colitis in mice by interacting with PDLIM3.

**Figure 6.**
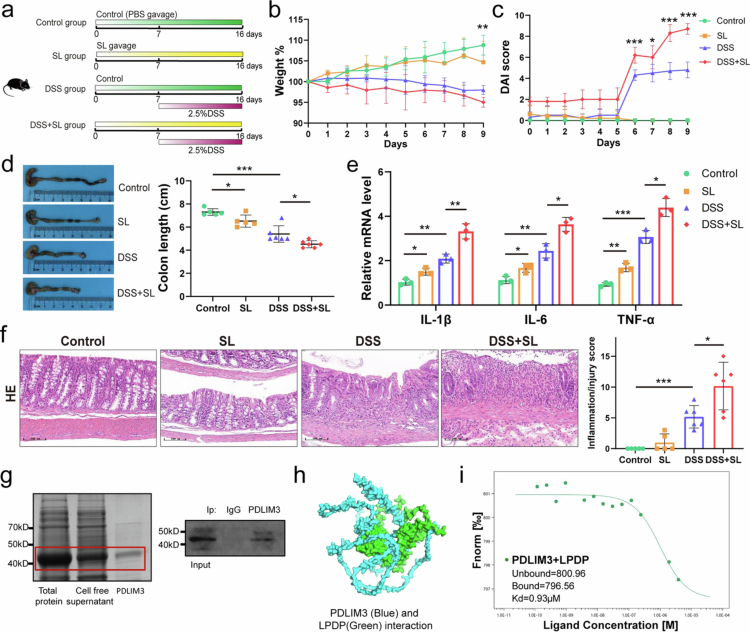
*Staphylococcus lentus* exacerbates DSS-induced colitis in mice through interacting with PDLIM3. (a) Experimental design. The mice were administered *Staphylococcus lentus* before DSS induction (*n* = 5 for control, SL; *n* = 6 for the DSS and DSS + SL). (b, c) Changes in body weight and DAI score. (d) Colon length (cm) on day 10 after DSS induction. (e) Relative mRNA level of IL-1β, IL-6, and TNF-*α* in colon. (f) Representative images of hematoxylin and eosin (H&E)-stained colon sections (left panel) and colon histopathological scores (right panel). Scale bar, 100 μm. (g) Purification of PDLIM3 and immunoprecipitation analysis of PDLIM3. (h) Predicted docking mode of LPDP and PDLIM3. PDLIM3 is displayed in blue, and LPDP is displayed in green. (i) Microscale thermophoresis analysis of the interaction between PDLIM3 and LPDP. SL *Staphylococcus lentus*, PDLIM3 PDZ and LIM domain 3, LPDP lysin motif peptidoglycan-binding domain-containing protein, DSS dextran sodium sulfate. Data are presented as the mean ± SEM; *P < 0.05, **P < 0.01, ***P < 0.001 by two-tailed one-way ANOVA.

Considering the increased abundance of *Staphylococcus lentus* in the KS and DSS + KS groups, we next explored the potential adverse effects of *Staphylococcus lentus* on the colon. *Staphylococcus lentus* was cultured and administered to the mice by gavage (Figure 6a). These results indicated that *Staphylococcus lentus* (SL) exacerbated DSS-induced colitis. The mice in the DSS + SL group showed greater weight loss, shorter colon length, and higher DAI (Figure 6b–d). H&E staining revealed a more severe inflammatory response (Figure 6e and 6f).

As mentioned earlier, the abundance of *Staphylococcus lentus* in the mice of the KS group was higher than that of control group. PDZ-binding motifs of PDLIM3, typically located in the extreme C terminus of the interacting protein, exhibit remarkable specificity (27). Considering the specificity of PDLIM3 binding to proteins and its important role in signal transduction, we assumed that there might be an interaction between proteins from bacteria such as *Staphylococcus lentus* and PDLIM3. We explored their interaction via co-immunoprecipitation after *Staphylococcus lentus* was cultivated and PDLIM3 was expressed in vitro (Figure 6g, Figure S7a). The results of the LC‒MS analysis showed that 809 proteins from *Staphylococcus lentus* could interact with PDLIM3 (Figure S7b). Only 145 proteins, most of them were enzymes, possess C-terminal sequences that PDZ domains can recognize (Table S4). The lysin motif (LysM) peptidoglycan-binding domain-containing protein (LPDP) was identified as the main protein that interacts with PDLIM3 and activates downstream signaling pathways, because it might be indispensable for the virulence of many pathogens. Therefore, LPDP was expressed in vitro (Figure S7c and d), and the interaction between PDLIM3 and LPDP was further confirmed by protein‒protein docking and microscale thermophoresis (Figure 6h and i, Figure S7e, f).

### 2.7 Tubuloside B alleviates colitis by blocking the interaction of PDLIM3 and LPDP

**Figure 7.**
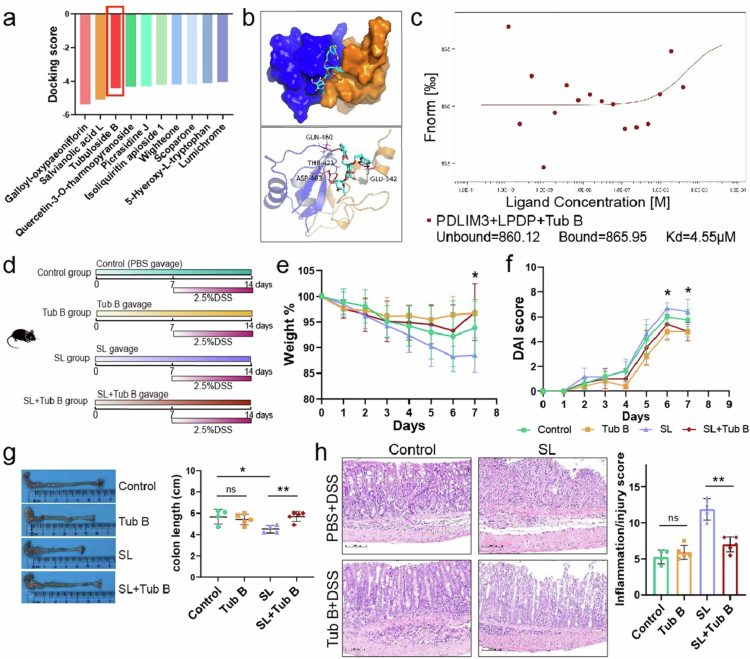
Blockade of the interaction between LysM peptidoglycan-binding domain-containing protein and PDLIM3 by tubuloside B alleviates SL-induced colitis in mice. (a) Molecular docking results of high-throughput screening based on the interaction of LPDP and PDLIM3. (b) Predicted docking model of the three-dimensional structure of tubuloside B and the PDLIM3/LPDP complex. PDLIM3 is displayed in yellow, LysM peptidoglycan-binding domain-containing protein is displayed in blue, and tubuloside B is displayed in green. (c) Microscale thermophoresis results for the binding of LPDP to PDLIM3 in the presence of tubuloside B. (d) The experimental design of DSS colitis model (*n* = 5 for the control and Tub B groups; *n* = 6 for the SL and SL + Tub B). (e, f) Body weight and DAI score. (g) Colon length (cm) on day 8 after DSS induction (*n* = 4 for the control, *n* = 5 for Tub B; *n* = 4 for SL, *n* = 5 for SL + Tub B). (h) Representative images of hematoxylin and eosin (H&E)-stained colon sections (left panel) and colon histopathological scores (right panel). Scale bar, 100 μm. LPDP, lysin motif peptidoglycan-binding domain-containing protein; PDLIM3 PDZ and LIM domain 3, DSS dextran sodium sulfate, Tub B tubuloside B. Data are presented as the mean ± SEM; *P < 0.05, **P < 0.01, ***P < 0.001, ns, not significant by two-tailed one-way ANOVA.

To block the interaction of PDLIM3 and LPDP, which activates downstream signaling pathways, we screened interface inhibitors through high-throughput screening from the Traditional Chinese Medicine (TCM) database (Figure 7a). We screened tubuloside B (Tub B) as an interface inhibitor to test its protective effect because of its easy availability. The interface inhibitory effect of Tub B was further confirmed by protein‒protein docking and microscale thermophoresis (Figure 7b and 7c). We subsequently explored the effect of Tub B on SL-exacerbated colitis (Figure 7d). There was no significant difference between the control and Tub B groups in terms of weight loss, DAI score, colon length, and inflammatory score after DSS administration, and Tub B obviously attenuated SL-exacerbated colitis, suggesting that Tub B itself did not alleviate DSS-induced colitis, but could attenuate SL-aggravated colitis (Figure 7e–h). Furthermore, our in vitro experiments demonstrated that Tub B alleviated SL-aggravated colitis by blocking the interaction of PDLIM3 with SL-derived protein (Figure S8a, b).

## 3. Discussion

The global increasing incidence of IBD, which parallels a westernized lifestyle and diet, has increased the number of experimental and clinical studies on diet-induced or diet-improved gut inflammation. Diet critically influences the gut microbial composition and function both in animal models and in humans.^4^ Our retrospective dietary survey revealed that IBD patients consumed more ultra-processed foods, especially fried and baked goods, than healthy individuals did, which was consistent with the results of a prospective cohort study from 21 countries. High consumption of fried foods and refined sweetened foods such as cakes and cookies increases the incidence of IBD.^7^ Excessive consumption of fried and baked foods can lead to high intake of unhealthy fats, particularly oils, during high-temperature cooking. In addition to harmful substances such as acrylamide produced during high-temperature cooking, POPs are also believed to pose health risks. POPs are various oxidized forms of phytosterols found in UPFs that have adverse effects on the liver and cardiovascular and nervous systems.^24^^,^^25^^,^^29^^,^^30^ The bioaccessibility of total POPs in simulated gastrointestinal digestion ranges from 19.08% to 49.29%.^30^ However, no studies have investigated the absorption rates of POPs in humans. Given the relatively low bioaccessibility of POPs, we hypothesize that they may impact the gut microbiota. KS was reported to account for the greatest proportion of the mixture of POPs.^26^^,^^31^ Therefore, our study aimed to focus on whether KS impaired the gut microbiota to promote the development of intestinal inflammation. The recommended intake of phytosterols is 2−3 g/d, and the oxidation rate of phytosterols in baked food was reported from 1.5% to 23.2%.^19^ We determined the KS dosage for the mouse model of colitis as 100 mg/kg based on previous reports, which corresponded to a relatively high intake and high oxidation rate of phytosterol.^29^^,^^30^ In this study, we demonstrated that KS aggravated experimental colitis with gut dysbiosis.

Our findings showed that KS was associated with increased abundance of *Staphylococcus* (*Staphylococcus lentus*), Corynebacterium_1 (*Corynebacterium stationis*), *Pseudogracilibacillus*, *Psychrobacter*, *Sporosarcina* and *Atopostipes*, and decreased abundance of *Ruminococcaceae_UCG-010*, *Clostridium_sensu_stricto_1*, *Clostridiales_vadinBB60_group,* and *Lachnospiraceae_UCG-006*. As an opportunistic pathogen, *Staphylococcus* was increased in IBD patients in database of human gut microbiota as well as mice after exposure to Western diet.^32^ *Staphylococcus lentus* increased the intestinal permeability and facilitated the development of disease in mice.^33^ *Staphylococcus lentus* and other potentially colitogenic commensal bacteria were reported to be bound by monoclonal IgA, preventing colitis development.^34^ Changes in abundance of *Corynebacterium_1* involved in restoring the mice intestinal barrier integrity impaired by high-sucrose or high-fat diet.^35^^,^^36^ The abundance of *Psychrobacter*, *Pseudogracilibacillus,* and *Staphylococcus* was increased in dyslipidemic mice treated with high-fat diet according to previous studies.^37^^,^^38^ *Sporosarcina* was also reported to be increased in high-fat, high-sugar diet, and related to intestinal inflammatory immune response.^39^^,^^40^ Meanwhile, we found *Ruminococcaceae_UCG-010*, *Clostridium_sensu_stricto_1*, *Clostridiales_vadinBB60_group*, and *Lachnospiraceae_UCG-006* were reduced in KS group. *Clostridium spp* is necessary in the metabolism of tryptophan into indole metabolites by fermenting amino acids, exerting positive effects on intestinal barrier function.^41^ The abundance of *Clostridium_sensu_stricto_1* was decreased in patients with CD.^41^ The *Clostridiales* order, which can produce butyrate, was enriched in healthy individuals and reduced in patients with obesity and UC,^42^^,^^43^ and *Clostridiales_vadinBB60* was decreased in experimental colitis.^44^ In addition, *Lachnospiraceae_UCG−006*, correlated with production of short-chain fatty acids, was increased in DSS-induced mice treated with prebiotics.^45^ However, when we exposed *Staphylococcus lentus* to KS in vitro, we found that KS not directly promote the growth of *Staphylococcus lentus*, indicating that the impact of KS on the gut microbiota was not limited to individual bacteria such as *Staphylococcus lentus*, but rather influenced the entire gut microecology. These findings are further consistent with previous studies and suggest that the effect of KS in UPFs on the gut microbiota contributes to the aggravation of colitis.

KS, a POP, is oxidized from *β*-sitosterol and relatively stable, and there are no studies supporting its further metabolism. POPs originate from the diet, autoxidation, and possibly enzymatic oxidation, and can be absorbed into the blood. It has been reported that 7-oxygenated products of sitosterol and campesterol are the main POPs in human plasma, ranging from 1 to 10 ng/ml.^46^ However, the intake of POPs ranges from 3.5 to 4.2 mg/d for the consumption of heated food,^20^ suggesting that most of the KS was excreted from the body. We conducted antibiotics and FMT experiments to investigate the impact of KS on DSS-induced colitis via the gut microbiota. We found that KS did not worsen colitis in mice when the gut microbiota was eliminated. However, DSS administration in mice resulted in a striking reduction in colitis symptoms and histological scores after the removal of the gut microbiota, and the mere removal of the gut microbiota alone could not conclusively demonstrate that KS exacerbated colitis via the gut microbiota. Interestingly, we discovered that the gut microbiota of the mice in the KS group aggravated intestinal inflammation. These findings further suggest that although KS is eventually metabolized out of the body, KS-induced dysbiosis can aggravate colitis. We also found that KS alone slightly affected the intestinal barrier, as manifested by a decrease in ZO−1 and Claudin−3 expression, which was also observed in the gut microbiota, such as an increased abundance of *Staphylococcus*. These findings suggest that high-dose KS could indeed alter the gut microbiota and disrupt the intestinal mucosal barrier.

Although RNA sequencing data showed the upregulation or downregulation of many genes, we detected and confirmed the upregulation of PDLIM3 using real-time PCR, Western blotting, and immunofluorescent staining. PDLIM3, also known as actin-associated LIM protein (ALP), contains an *N*-terminal post-synaptic density protein, Drosophila disc large, and zonula occludens-1 protein (PDZ) domain and a C-terminal Lin11, ISL-1, and Mec-3 (LIM) domain. It plays an important role in the cytoskeleton and signal transduction.^27^ PDZ domains in PDLIM are generally conserved domains that mediate specific protein‒protein interactions. PDZ domains first interact with short peptides, called PDZ-binding motifs, and then LIM domains activate downstream signaling pathways, mediating diverse cellular functions, such as cell‒cell junctions; the recognition of immune cells; and the control of proliferation, differentiation, and cellular migration.^28^ PDLIM3 is expressed mainly in skeletal, cardiac and smooth muscle, where it is involved in the organization of actin‒filament arrays in muscle cells.^47^ Studies have demonstrated that PDLIM3 can activate the p38MAPK pathway and regulate the differentiation and proliferation of skeletal muscle satellite cells and is even correlated with the prognosis and development of various diseases.^27^^,^^48^ However, there has been no study of the relationship between PDLIM3 and colitis. In our study, PDLIM3 was highly expressed in the intrinsic muscular layer of the colon but poorly expressed in the mucosal layer in normal mice. Patients with increased intake of fried and baked food presented increased PDLIM3 expression in colonic tissue, especially in the intercellular matrix of the mucosal layer. In addition, PDLIM3 was upregulated in the DSS group, and KS resulted in increased PDLIM3 expression in intestinal mucosal cells and the mesenchyme, which was consistent with the findings in UC patients. The activation of the p38MAPK/NF-κB pathway may result in the production of proinflammatory chemokines and cytokines.^49^ We also observed the activation of the p38MAPK/NF-κB pathway in parallel, which suggested that the activation of p38MAPK by PDLIM3 increased inflammation and intercellular components. Nevertheless, we found that PDLIM3 was not upregulated after removal of the gut microbiota, which suggested that the upregulation of PDLIM3 and activation of p38MAPK/NF-κB were dependent on the gut microbiota. PDZ-binding motifs of PDLIM3, typically located in the extreme C terminus of the interacting protein, exhibit remarkable specificity.^27^ PDLIM3 proteins can be classified into three major types based on their C-terminal peptide sequences: -Xaa-(Ser/Thr)-Xaa- *ψ* (class I); -Xaa-*ψ*-Xaa-*ψ* (class II); and -Xaa-(Asp/Glu)-Xaa-*ψ* (class III), where Xaa is any amino acid and where *ψ* is a hydrophobic residue. Considering the specificity of the PDZ domain with typical C-terminal peptide sequences and significant alterations in the abundance of *Staphylococcus lentus*, we assumed that there might be interactions between *Staphylococcus lentus*-derived proteins and PDLIM3. Yi CR et al reported the interaction between proteins from bacteria and the PDLIM family.^50^ Effector proteins OspE from *Shigella fexneri* could bind to PDLIM7 during infection and activate isoforms of protein kinase to enhance bacterial spread.^50^ This suggests that the aggravation of intestinal inflammation induced by KS in mice is related to the interaction between *Staphylococcus lentus* and PDLIM3.

We found that *Staphylococcus lentus* aggravated DSS-induced colitis, and proteins from *Staphylococcus lentus* were shown to interact with PDLIM3 via co-immunoprecipitation and LC–MS. According to the C-terminal sequences and functions of proteins, we identified the Lysin motif (LysM) peptidoglycan-binding domain-containing protein (LPDP) as the main protein that interacts with PDLIM3. LysM is widely present in proteins of both eukaryotes and prokaryotes, and possesses highly conserved carbohydrate-binding capabilities. LysM domains from many LysM-containing proteins retain their ability to bind peptidoglycan, chitin, or lipooligosaccharides even if they are removed from their natural context. It is indispensable for many pathogens to exert virulence and immunosuppressive responses.^51^ SpA, a surface protein of *Staphylococcus_aureus*, also contains a LysM domain, which significantly enhances cross-wall targeting.^52^ The LysM domain of *Staphylococcus_aureus* can bind to fibrinogen, fibronectin, and vitronectin to exert adhesion and virulence.^53^ We confirmed the interaction of PDLIM3 and LPDP via microscale thermophoresis. Although proteins from other bacteria might interact with PDLIM3, our data suggest that proteins from the gut microbiota could interact with PDLIM3 and then activate the inflammatory signaling pathway to promote colitis.

IBD is a recurrent and intractable inflammatory disease that urgently requires new treatment strategies. Considering the potent therapeutic efficacy and limited side effects, traditional Chinese medicine (TCM) has recently received increasing attention in IBD therapy. In this study, we selected tubuloside B by high-throughput screening of the TCM database. Tubuloside B is a phenylethanoid isolated from the stems of *Cistanche tubulosa*, a traditional Chinese medicine named “Rou Cong Rong”.^54^ Tubuloside B has been reported to have antioxidation, hepatoprotection, and neuroprotective effects without general toxicity or genetic toxicity.^54-57^ Recent studies reported that tubuloside B has an anti-inflammatory effect by inhibiting M1 macrophage activation.^58^ We demonstrated the role of tubuloside B in blocking the interaction of PDLIM3 and LPDP to inhibit gut dysbiosis-induced colitis, providing a new candidate for colitis treatment. Despite this, further experiments are needed to explore the efficacy and specificity of tubuloside B.

However, our study has certain limitations. First, although we deliberately included IBD patients with a short disease duration and confirmed that they had no significant changes in dietary habits in the year prior to the survey, recall bias still existed. Therefore, the calculated intake of KS may not reflect the actual intake. However, during our actual survey, we found that the dietary habits of IBD patients remained generally consistent for years before the onset of the disease, which further strengthens the credibility of our results. Second, this study solely focuses on *Staphylococcus lentu*s. It is possible that other bacterial proteins also interact with PDLIM3. In addition, the effects of other POPs on the gut and microbiota also need further exploration.

**Figure 8.**
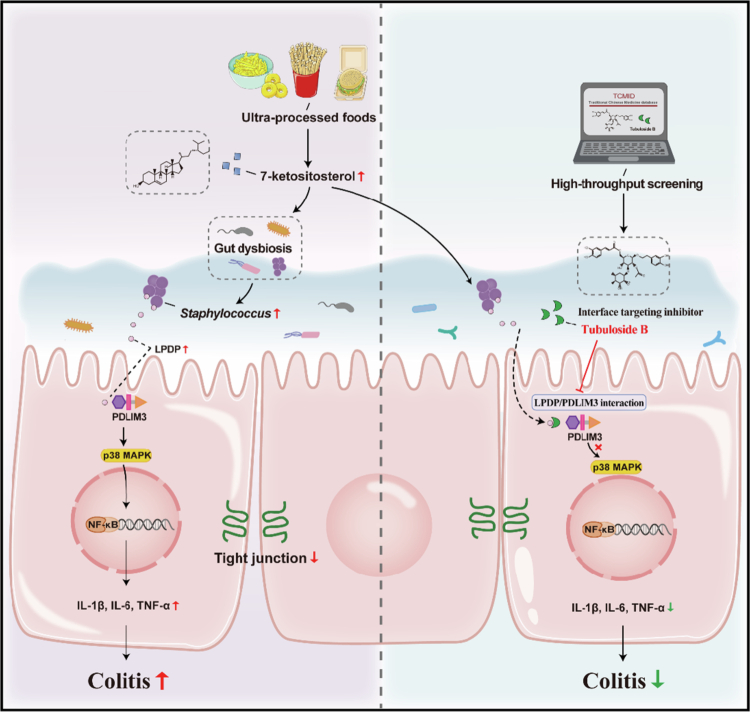
Schematic summary of the role of KS-induced colitis. Excessive intake of ultra-processed foods means increased exposure to KS. KS exacerbated DSS-induced colitis in a gut microbiota-dependent manner and resulted in gut dysbiosis, especially increasing the abundance of *Staphylococcus lentus*. *Staphylococcus lentus*-derived LPDP could interact with PDLIM3 and activate the p38MAPK/NF-κB signaling pathway, and the binding interfaces could be blocked by tubuloside B, a Chinese herbal extract selected by high-throughput screening, to ameliorate colitis. KS 7-ketositosterol, DSS dextran sodium sulfate, LPDP, lysin motif peptidoglycan-binding domain-containing protein, PDLIM3 PDZ and LIM Domain 3.

In summary, our study highlights the impairment of the intestine as a result of KS exposure in mice model. We demonstrated that the upregulation of PDLIM3 and downstream p38MAPK/NF-κB pathway activation are dependent on gut dysbiosis. Proteins from bacteria such as *Staphylococcus lentus* could interact with the PDZ domain of PDLIM3. We identified the adverse effects and potential mechanisms of KS, providing a possible dietary strategy for preventing the development of IBD as well as a potential therapeutic method for treating colitis (Figure 8).

## 4. Experimental section/methods

*Human study design*: Eligible patients diagnosed with UC or CD were recruited at Tianjin Medical University General Hospital (Tianjin, China) from September 2021 to July 2023. Feces samples were collected for fecal calprotectin detection, and colonic samples were taken for staining during the first colonoscopy visit without receiving any medication or biologics. All samples were snap-frozen and stored at −80 °C. Healthy controls, ensuring age and gender matched, were randomly recruited via public advertising. Patients with UC who had a Mayo score ≥ 3 or patients with CD who had a Crohn's Disease Activity Index (CDAI) > 150 were diagnosed as being in the active stage based on the clinical manifestations, radiology, endoscopy, and histology. The exclusion criteria were as follows: a history of colorectal surgery; pregnancy or lactation; being complicated with Cytomegalovirus or *Clostridium difficile* infectious enteritis; having psychiatric disorders or unconsciousness; being unable to complete the questionnaire independently; and being complicated with severe cardiovascular, hepatic, renal, or respiratory diseases. To reduce the survey bias caused by possible dietary adjustment following IBD diagnosis, we also excluded patients with a disease course of >2 y.

In cohort 1, dietary intake information of the participants was collected using a fine-tuned, semiquantitative food frequency questionnaire (FFQ) with 15 food groups (Table S2). IBD patients were asked about dietary intake information 1 y before the disease, and healthy controls were asked about their dietary situation in the last 1−2 weeks. All the subjects were confirmed to have experienced no significant dietary changes in the year prior to the survey. To reduce bias, we referenced the food portions in the Dietary Guidelines for Chinese Residents when describing the food consumption of the participants. Furthermore, we limited the number of investigators to no more than 2 to minimize bias caused by differences in understanding among investigators, and we ensured that all investigators were trained before the survey. POP intake was calculated based on the consumption of cooking oil and fried and baked foods per week because these foods are rich in oils and constitute the main source of POPs. Therefore, the POP level was approximately calculated because other plants containing POPs were not included such as cereals, nuts, legumes, vegetables, fruits, and tubers. In addition, the POP contents reported in different studies differ slightly. POPs were calculated as 3.3 μg/g fat in baked foods.^19^ Nevertheless, POPs in fried foods and cooking oils have been inconsistent across different reports. We uniformly calculated POPs as 1.2 μg/g fat in fried foods and cooking oils.^59^^,^^60^ KS accounts for approximately 50% of POPs according to the literature.^31^ The characteristics of the participants are presented in Table S1.

Considering the bias of dietary survey and estimation, in cohort 2, we collected blood samples from healthy controls (*n* = 20) and patients with new-onset UC (*n* = 20). KS concentration in serum was detected to confirm its accuracy and analyze its correlation with disease activity. This study was approved by the Clinical Research Ethics Committee of Tianjin Medical University General Hospital (No. IRB2024-YX-152-01).

*Detection of fecal calprotectin*: Fecal calprotectin data were obtained from Tianjin Medical University General Hospital and measured using an enzyme-linked immunosorbent assay (ELISA) kit. Fecal samples from IBD patients and healthy controls were stored at −20 °C until analysis. Prior to testing, 100 mg of each sample was suspended in a buffer solution and centrifuged at 10,000 × g for 10 min to collect the supernatant. In accordance with the manufacturer’s protocol, 100 µl of the supernatant was added to precoated microtiter wells, followed by the addition of a secondary antibody. After incubation, a substrate mixture was added, and the reaction was terminated with a stop solution. The optical density was read at 450 nm, and the concentrations of calprotectin were determined using a standard curve, with elevated levels indicating intestinal inflammation.

*KS concentration detection*: Serum levels of KS were measured using ELISA Kit (MM-928634O1; Jiangsu MeiMian Industry Co. Ltd., China). Blood samples from healthy controls and patients with new-onset UC were frozen and thawed three times at –20 °C and then filtered through glass fibers. Serum samples and enzyme reagents were added to every plate and incubated for 60 min at 37 °C. Washing solution was then added for 30 s, which was repeated 5 times. Chromogenic reagent was added, and the OD was measured at 450 nm with a microplate reader. The content of KS in the samples was calculated via a standard curve.

*KS synthesis*: 7-Ketositosterol was synthesized from *β*-sitosterol by Birdo Tech (Xi’an, China). A solution of *β*-sitosterol (82.8 g, 0.2  mmol, 1.0 eq) and CuCl(OH)*TMEDA (18.56 g, 0.05  mmol, 0.2 eq) in CH_2_Cl_2_ (1.6 L) and MeOH (400 mL) was added to t-BuOOH (30% aqueous, 300 g, 1.0 mol, 5 eq) at room temperature. The reaction was stirred at room temperature for 48 h. After completion, the solvent was removed under reduced pressure. The resulting mixture was diluted with NaHSO_3_ aqueous solution (200 mL) and then extracted with EtOAc (200 mL*3). The organic layer was washed with brine, dried and concentrated. The residue was purified by flash column chromatography and eluted with EtOAc/petroleum ether (1:2–1:1) to yield KS (33.0 g, 40.9%) as a white solid.^^61^^ The identity of the final product was confirmed by liquid chromatography–mass spectrometry (LC–MS) with 95.922% purity, and the remaining 4.078% was *β*-sitosterol.

*Animal experiments and treatments*: Female C57BL/6 mice (7 weeks old) were purchased from the Laboratory Animal Co. Ltd. (Wuhan, China) and housed under specific-pathogen-free conditions with air filtration (22 ± 2 °C) and free access to standard rodent food and sterilized water. All the animal experiments were performed in accordance with the Tianjin Medical University animal utilization protocols and approved by the Management and Use of Laboratory Animals Committee (No. 202210095).

To evaluate the effect of the gut microbiota, the mice were administered antibiotic cocktails mixed with 1 g/L ampicillin, 1 g/L neomycin, 1 g/L metronidazole, and 500 mg/L vancomycin to eliminate the gut microbiota. The mice were then treated differently according to the purpose.

DSS (dextran sodium sulfate; molecular weight 36–50 kDa; MP Biomedicals) was dissolved in sterilized water to a concentration of 2.5%. The mice were randomly divided into four groups: the control group, in which they received PBS (days 0–21); the DSS model group, in which they received PBS (days 0–21) and DSS application on days 15–21; the 7-ketositosterol group (KS), in which they received 7-ketositosterol application from days 0–21; and the 7-kitositosterol treatment group (DSS + KS), in which they received 7-ketositosterol (days 0–21) and DSS added on days 15–21. KS (100 mg/kg/d, dissolved in water) was administered daily by gavage (200 μl/mouse). The dose was chosen based on previous studies of POPs.^29^^,^^62^ The weights of the mice were recorded daily after DSS induction, and the percentage of the weight at day 14 was defined as 100%. Fresh fecal samples were collected 2 d before the mice were sacrificed and stored in a −80 °C refrigerator.

Trinitro-benzene-sulfonic acid (TNBS) was used to induce colitis. The mice were randomly divided into 4 groups: the control, KS, TNBS, and TNBS + KS groups. On the 11th day, the mice in the TNBS groups (TNBS and TNBS + KS groups) were subcutaneously injected with 150 μl of a 1% TNBS solution in a mixture of acetone and olive oil (4:1 in volume). Seven days later, the mice in the TNBS groups (TNBS and TNBS + KS groups) received a 100 μl enema of a 2.5% TNBS solution (1:1 in volume of 5% TNBS and ethanol), while mice in the control groups (control and KS groups) were administered a 100 μl enema of 50% ethanol. In addition, the mice in the control groups (control and TNBS groups) and KS groups (KS and TNBS + KS groups) were treated with PBS or KS (200 μl, 100 mg/kg), respectively, from the 1 st to 21 st day as described previously. Mice were sacrificed on the 21 st day to evaluate inflammation.

For Abx mouse model, 36 female C57BL/6 mice were randomly divided into five groups: control group (*n* = 6), KS group (*n* = 6), DSS group (*n* = 8), DSS + KS group (*n* = 8), Abx + DSS + KS group (*n* = 8). The mice in the first four groups were treated as previously described without antibiotics, while mice in the Abx + DSS + KS group received antibiotic cocktails mixed with 1 g/L ampicillin, 1 g/L neomycin, 1 g/L metronidazole, 1 g/L vancomycin, and 500  mg/L vancomycin for 21 d. KS was administered by gavage from days 0–21, with DSS added from days 15–21.

For the fecal microbiota transplantation model, 200 mg of fresh feces was collected from the control and KS groups and placed in 5 mL of sterile PBS solution with 0.05% cysteine hydrochloride as the fecal mixture. The fecal mixture was shaken for 3 min and left for 2 min on ice under anaerobic conditions. The supernatant was collected in a Hungate anaerobic tube and stored in a refrigerator (−80 °C). Ten 8-week-old C57BL/6 mice were given antibiotic cocktails in the drinking water for 5 d to eliminate the pre-existing intestinal microbiota and then randomly divided into two groups: the FMT-Con group (gavaged with fecal supernatant from the control group) and the FMT-KS group (gavaged with fecal supernatant from the KS group). Fecal supernatant (200  μl) was given to each mouse by gavage three times a week for 21 d. At the end of the period, DSS was given for 7 d, and no additional fecal supernatant was given during the administration of DSS.

For the *Staphylococcus lentus*-aggravated colitis model, 22 female C57BL/6 mice were randomly divided into four groups: the control group (*n* = 5), the SL group (*n* = 5), the DSS group (*n* = 6), and the DSS + SL group (*n* = 6). All the mice received antibiotic cocktails for 7 d to eliminate the gut microbiota. The mice in the SL and DSS + SL groups were subsequently given pure *Staphylococcus lentus* (10^9^ CFU) by gavage from days 1–16. Considering that *Staphylococcus* is not a well-established pathogen, there is limited research on this pathogen, and the absence of a gut microbiota can alleviate symptoms of colitis, we added 2 d to the routine 7-d DSS treatment to ensure a more successful colitis model. Therefore, the mice in the DSS and DSS + SL groups received DSS from days 8–16.

For the tubuloside B-treated model, tubuloside B (CAS: 112516-04-8, purity > 98.0%) was obtained from Hubei Deao Chemical Research Pharmaceutical Technology Co., Ltd., China. Twenty-two female C57BL/6 mice were randomly divided into four groups: the control group (*n* = 5), the Tub group (*n* = 5), the SL group (*n* = 6), and the SL + Tub group (*n* = 6). Similarly, all the mice received antibiotic cocktails for 7 d to eliminate the gut microbiota. Afterwards, tubuloside B (10 mg/kg) was administered to the mice in the Tub and SL + Tub groups by gavage for 14 d, and *Staphylococcus lentus* (10^9^ CFU) was given to the mice in the SL and SL + Tub groups for 14 d. All the mice were subsequently given DSS from days 8–14.

The mice were subsequently euthanized. The colons were collected and cut longitudinally. The proximal part of the colon was quickly frozen in liquid nitrogen and stored at −80 °C for mRNA and protein expression. The distal part was rolled as a “Swiss roll” and fixed with a 4% paraformaldehyde solution for staining.

*Bacterial strain and culture conditions*: *Staphylococcus lentus* was obtained from BeNa Culture Collection (BNCC, China). It was cultured on sterilized Luria-Bertani Agar (Sigma, USA) at 37 °C and further cultured in LB Broth (HOPEBIO, China) at 37 °C. To investigate the effect of KS on the growth of *Staphylococcus lentus*, 4.29 mg of KS was dissolved in 10.00 mL of anhydrous DMSO to prepare a 1  mM stock solution, which was then diluted to 10, 1, and 0.1 μM, respectively. These diluted solutions were added to culture medium containing *Staphylococcus lentus*. The growth of SL was observed after 4, 8, 12, 16, 24, and 28 h of incubation, and a growth curve was plotted.

*Cell culture and treatment*: The human colonic epithelial cell line Caco−2 was grown in Eagle's minimum essential medium (MEM) supplemented with **1%** nonessential amino acids, 1% penicillin and streptomycin and 20% fetal bovine serum (FBS) at 5% CO2 and 37 °C. When the epithelial cells reached approximately 70% confluence in six-well plates, they were starved with MEM containing 1% nonessential amino acids, 1% penicillin and streptomycin, and 0.5% FBS for 12 h. Then, Caco-2 cells were treated with or without *Staphylococcus lentus* liquid supernatant (SL) diluted to half of the original concentration and 100  μg/mL tubuloside B (Tub B) for 24 h.

*Histology and immunohistochemistry*: The colon tissues were first fixed in 4% neutral-buffered formalin and then processed through dehydration in graded ethanol (70%–100%), xylene clearing, and paraffin embedding. Colon tissues were then cut into serial sections (4 μm thick). Following deparaffinization and rehydration, H&E staining was performed. H&E-stained sections were imaged with a light microscope SZX16 (Olympus, Japan) for analysis of inflammatory infiltration.

*Immunohistochemical staining* was performed as follows: After antigen retrieval in sodium citrate buffer (pH 6.0) and blocking with 5% bovine serum albumin (BSA) (30 min), the tissue sections were incubated with an anti-MUC2 primary antibody (Santa Cruz Biotechnology, Inc.) overnight at 4 °C. After diaminobenzidine development and hematoxylin counterstaining, the slides were mounted with resin-based medium. MUC2 expression was quantified by counting positively stained cells per crypt, with 100 randomly selected crypts per section. Digital images were acquired at​​ ​​400× magnification (DM5000 B, Leika, Germany) from five representative fields, ensuring  that ≥100 crypts were analyzed per animal.

*Periodic acid Schiff (PAS) staining*: PAS staining was performed on paraffin-embedded colonic sections through sequential steps: deparaffinization in xylene, rehydration through graded alcohols, oxidation in 1% periodic acid (Sigma–Aldrich, 10 min) and Schiff reagent (Sigma–Aldrich, 40 min). Hematoxylin counterstaining (2−5 min) was used to visualize the nuclei, and rigorous PBS washes were used to ensure staining specificity.

*Immunofluorescent staining*: Paraffin-embedded tissues were subjected to deparaffinization, hydration, and antigen retrieval. Nonspecific binding was blocked using a 5% BSA solution. Colon tissues were then exposed to specific anti-ZO-1 and anti-PDLIM3 antibodies (Boster Biologicals Technology) overnight at 4 °C. After that, the sections were incubated with the fluorochrome-conjugated secondary antibody IgG H&L (Boster Biologicals Technology) at room temperature for 1 h. DAPI (49,6-diamidino-2-phenylindole) was applied to stain the nucleus. The addition of the secondary antibody and nuclear staining were performed in the dark as much as possible. A PBS solution was used to wash the sections at each step. Fluorescence images were captured using a fluorescence microscope (DM5000 B; Leica, Germany), and ZO-1-positive cells were analyzed.

*Fecal DNA extraction and Staphylococcus quantification*: Human fecal DNA was extracted using QIAamp DNA Stool Mini Kit (QIAGEN, Germany). A total of 180−220 mg of fecal samples was weighed and transferred to a microcentrifuge tube. The tube was placed on ice. InhibitEX buffer was added to each fecal sample, which was subsequently vortexed until the samples were thoroughly homogenized. Then, the suspension was heated for 5 min at 70 °C and centrifuged for 1 min. The supernatant was added to a microcentrifuge tube containing proteinase K, and AL buffer was added. The samples were then incubated at 70 °C for 10 min. The lysate supplemented with 200 μl of ethanol was applied to the QIAamp spin column and centrifuged for 1 min. The QIAamp spin column was further placed in a new collection tube. Five hundred  microliters of AW1 and AW2 buffers were added to the QIAamp spin column sequentially, followed by centrifugation for 1 and 3 min, respectively. The QIAamp spin column was finally placed in a new microcentrifuge tube, and 200 μl of Buffer ATE was added onto the QIAamp membrane for incubation and centrifugation to elute the DNA. The yield was quantified by UV absorbance. qPCR was then conducted to quantify the level of *Staphylococcus* in the fecal samples. The primers used for the analysis were as follows: *Staphylococcus* (forward primer: GCTGGCGGCGTGCCTAATACA; reverse primer: GTGTCTCAGTTCCAGTGTGGCC) and universal bacteria (forward primer: GCAGGCCTAACACATGCAAGTC; reverse primer: CTGCTGCCTCCCGTAGGAGT).

*ELISA assay*: After full homogenization, the mouse colon tissue homogenates were centrifuged at 5000 × g for 5–10 min, and the supernatants were collected. The levels of IL-1β, TNF-*α,* and IL-6 were assessed using ELISA kits in accordance with the manufacturer's instructions. Standard, zero standard, blank, and sample wells were established. The optical density (OD) at 450 nm was measured for each well. A standard curve was plotted by relating the known standard concentrations to their corresponding OD values, and the sample concentrations were calculated accordingly.

*Real-time PCR analysis*: Total RNA was isolated from colon tissues was extracted with TRIzol reagent (Invitrogen, La Jolla, CA), with additional lithium chloride purification required for DSS-treated samples. Reverse transcription was performed using the TIAN Script RT Kit (TIANGEN, Inc., Beijing, China) following the instructions. Real-time PCR amplification was carried out in triplicate using TaqMan Gene Expression Master Mix and primers (GENEWIZ, Inc., Beijing, China) with predesigned primers (Table S3) targeting TNF-*α*, IL-1β, IL-6, PDLIM3, and glyceraldehyde-3-phosphate dehydrogenase (GAPDH). Gene expression fold changes were calculated by 2−∆∆CT method with GAPDH normalization.

*Western blotting*: The colonic tissues were adequately homogenized in radioimmunoprecipitation assay (RIPA) buffer supplemented with the protease inhibitor phenylmethanesulfonyl fluoride (PMSF) (Solarbio, Beijing, China) at 4 °C. Afterwards, the protein concentration was determined via a bicinchoninic acid protein assay (Thermo Scientific, Inc.). The protein samples were boiled with a certain volume of loading buffer, loaded and separated using SDS polyacrylamide gel electrophoresis and then blotted onto a polyvinylidene fluoride (PVDF) membrane (Invitrogen, Carlsbad, CA, USA). The membranes were subsequently incubated overnight at 4 °C with primary antibodies against CLND−3 (rabbit anti-mouse; Affinity Biosciences), ZO-1 (rabbit anti-mouse; Proteintech Group), PDLIM3 (rabbit anti-mouse; Proteintech Group), p38 (anti-mouse monoclonal antibody, Affinity Biosciences), *p*-p38 (rabbit anti-mouse; Affinity Biosciences), p65 (anti-mouse monoclonal antibody, Affinity Biosciences), *p*-p65 (rabbit anti-mouse; Affinity Biosciences), IκBα (rabbit anti-mouse; Affinity Biosciences), and *p*-IκBα (rabbit anti-mouse; Affinity Biosciences) and then incubated with HRP-conjugated secondary antibodies (Beyotime Biotechnology). The intensity of the Western blot images was quantified by ImageJ.

*Plasmid construction and protein expression*: Mouse PDLIM3 and LPDP cDNA was obtained according to its whole-genome sequence. The cDNA sequences of PDLIM3 and LPDP were subsequently cloned into the PEt-28a ( + ) and PEt-28a ( + )-sumo vectors, respectively, with the XhoI and BamHI enzymes used as restriction sites. The vectors were transfected into BL21 *Escherichia* *coli*, which were subsequently grown in LB media supplemented with kanamycin at 37 °C. Then, 0.4 mM isopropyl *β*-D-thiogalactoside (IPTG, Sigma) was added to the bacterial mixture to induce protein expression until the OD600 reached 0.6, and the bacterial mixture was subsequently incubated at 16°C for 12 h. The bacterial cells were collected via centrifugation at 4500 rpm for 15 min, and protein expression was detected by electrophoresis. Next, the bacterial cells were broken using a high-pressure cell disrupter to obtain total protein. The supernatant from the bacterial lysis was collected after centrifugation and loaded into chromatographic column with NI-NTA agarose media. PBS buffers containing sequential concentrations of imidazole (20 mM/50 mM/250 mM, pH 8.0) were used to obtain the purified fusion protein. The purified fusion protein was condensed, and the tag was removed by Sumo protease.

*Coimmunoprecipitation*: *Staphylococcus lentus* was cultivated and collected. A PBS solution was added to obtain a bacterial mixture. *Staphylococcus lentus* was disrupted by a high-pressure cell disrupter and centrifuged. The supernatant was then collected for further co-immunoprecipitation. Purified PDLIM3 was incubated with the *Staphylococcus lentus* supernatant overnight at 4 °C. Protein A + G Agarose beads (Beyotime, China) were added to the quantified proteins to eliminate non-specific binding proteins, which were then discarded after centrifugation. Next, the PDLIM3 antibody was added, and the mixture was allowed to shake overnight at 4 °C. Protein A + G Agarose beads were then added to bind the antigen‒antibody complex. The sediment was collected and washed with prechilled PBS before Western blotting.

*Protein‒protein docking*: The entry ID of PDLIM3 was O70209•PDLI3_MOUSE, which was obtained from UniProt (https://www.uniprot.org/), and the crystal structure was downloaded. The entry ID of the LysM peptidoglycan-binding domain-containing protein (LPDP) was A0A8I0UA09•A0A8I0UA09_MAMLE, and the three-dimensional structure was created via SWISS-MODEL (http://swissmodel.expasy.org/) according to the amino acid sequence. The protein docking was performed via GRAMM software with PDLIM3 as a receptor and LPDP as a ligand, and the docking results were analyzed via PDBePISA (https://www.ebi.ac.uk/msd-srv/prot_int/cgi-bin/piserver) to find the best docking model. Interface inhibitors were subsequently selected through high-throughput screening from the Traditional Chinese Medicine (TCM) database according to the interface of the PDLIM3/LPDP complex.

*Virtual screening of the Traditional Chinese Medicine Database*: Virtual screening was performed using the TCM Database@Taiwan (http://tcm.cmu.edu.tw/), a comprehensive database containing 17,459 TCM-derived small molecules with experimentally validated chemical structures and pharmacological properties. The screening was conducted using Schrödinger software suite (Schrödinger, LLC, New York, NY) with a three-stage hierarchical docking protocol. Initially, the PDLIM3/LPDP complex interface was defined as the target binding site, and receptor grids were generated using the Receptor Grid Generation module in Maestro. The virtual screening workflow was then implemented in three sequential stages: (1) High-throughput virtual screening (HTVS) docking of all 17,459 compounds, with the top 10% (approximately 1,746 compounds) selected based on docking scores; (2) Standard precision (SP) docking of the selected compounds from stage 1, with the top 10% (approximately 175 compounds) advanced to the final stage; and (3) Extra precision (XP) docking for the most promising candidates, with final ranking based on XP docking scores and binding interactions. The compounds were selected based on favorable docking scores, specific interactions with key residues at the PDLIM3/LPDP interface, compliance with Lipinski's Rule of Five, and commercial availability for experimental validation.

*Microscale thermophoresis*: The binding affinity was detected with Monolith NT.115 (NanoTemper Technologies, Monolith, Germany). The PDLIM3 protein was fluorescently labeled with a complex Monolith His-Tag Labeling Kit (NanoTemper Technologies, Germany). LPDP protein or tubuloside B was dissolved in MST buffer (50  mM Tris-HCl, pH 7.4; 150  mM NaCl; 10  mM MgCl_2_). The samples were loaded into NT.115 glass capillaries. Kd was calculated using the MO Affinity Analysis v2.3 software.

*16S rRNA amplicon sequencing*: Fresh feces from the mice were collected for the extraction of total fecal bacterial DNA using a QIAamp® Fast DNA Stool Mini Kit (QIAamp, Germany). The 16S rRNA gene sequencing procedure was carried out by the Bioyigene Biotechnology Co. Ltd. (Wuhan, China) using an Illumina HiSeq platform. We used a Thermo Scientific NanoDrop 2000 UV microspectrophotometer and 1.2% agarose gel electrophoresis for quality control. Specific primers (341F: 5’-ACTCCTACGGGRSGCAGCAG−3’, 806R: 5’-GGACTACVVGGGTATCTAATC−3’) were prepared for PCR amplification using TransStart® FastPfu high-fidelity DNA polymerase, and product purification was finished by Vazyme VAHTSTM DNA Clean Beads. First, the sequencing library was constructed and subjected to quality inspection. Next, the libraries were sequenced using Illumina NovaSeq. The library sequences were quality filtered, trimmed, denoised, and merged, and then the chimeric sequences were identified and removed to analyze the abundance, diversity, and composition of the gut microbiota. Greengenes database was used to perform taxonomic assignment. Microbiome bioinformatics, including analyses of the composition of the gut microbiota and alpha and beta diversity indices, was performed with QIIME 2 2019.4. R and linear discriminant analysis effect size (LEfSe) were used to analyze different species and principal components.

*RNA sequencing*: Total RNA from colon cells was extracted and purified. Double-strand cDNA was synthesized using mRNA as a template and then repaired and purified. Finally, PCR amplification was performed to obtain the library. The library was sequenced using DNBSEQ-T7. The gene expression level was calculated and followed by alternative splicing analysis, novel transcript prediction, and gene structure optimization. Then the differentially expressed genes were processed using DESeq2 or EdgeR. GO functional enrichment and KEGG pathway analyses were carried out using clusterProfiler. We selected the gene sets with high expression levels and significant differences between groups.

*LC‒MS analysis*: The obtained fusion protein mixtures were identified through LC‒MS analysis. Proteins were extracted by denaturation, reduction, alkylation, and digestion. Mass spectrometry data were collected using a Q Active HF mass spectrometer combined with a liquid phase (UltiMate 3000 RSLCnano). The protein sample was dissolved in loading buffer and then passed through the analysis column. An analytical gradient was established in two mobile phases (mobile phase A: 0.1% formic acid and 3% DMSO; mobile phase B: 0.1% formic acid, 3% DMSO, and 80% ACN). The flow rate of the liquid phase was 300 nL/min. The data were collected in data dependent acquisition (DDA) mode and retrieved using MaxQuant (V1.6.6). The databases utilized for retrieval were the customized proteome reference database 231110101 and the proteome reference database for *Staphylococcus lentus* in UniProt. The search results were screened based on the 1% fault discovery rate (FDR) for subsequent quantification and comparison.

*Statistical analysis*: Prism 9 (GraphPad Software Inc., La Jolla, CA) and SPSS 24.0 (SPSS, Chicago, IL, USA) were used for all data analysis. The difference between groups was assessed using a *t*-test. One-way analysis of variance (ANOVA) was used for multiple samples. Post hoc Tukey’s comparisons were used to test for pairwise differences. Tukey’s test was used to generate a critical value to control the familywise error rate. Correlation analysis was conducted using a Spearman correlation coefficient. Multivariate logistic regression analysis was performed to assess associations between different exposures and disease. The measurement data are presented as the mean ± SEM, and nonnormally distributed continuous variables are expressed as the median, with *P-*value of < 0.05 was considered statistically significant.

## Supplementary Material

Supplementary material Ultra-processed foods sourced from 7-ketositosterol aggravate colitis through gut dysbiosis induced-PDLIM3 activation.

## Data Availability

The data in this article have been deposited to the NCBI’s GEO. The RNA-seq and 16S rRNA-seq data are available at: https://www.ncbi.nlm.nih.gov/geo/query/acc.cgi?acc=GSE299083
