# Supplementary material for: Ultra-processed foods sourced 7-ketositosterol aggravates colitis through gut dysbiosis induced-PDLIM3 activation
Source: Gut Microbes. 2025 Nov 24;17(1):2587980. doi: 10.1080/19490976.2025.2587980 (PMC12931730; doi:10.1080/19490976.2025.2587980)
Supplement: Supplementary material — Ultra-processed foods sourced from 7-ketositosterol aggravate colitis through gut dysbiosis induced-PDLIM3 activation. [file KGMI_A_2587980_SM1755.docx]

**Table S1. Characteristics and dietary information of healthy individuals and IBD patients.**

|  | HD (n=296) | UC (n=158) | *p* | CD (n=100) | *p* |
| --- | --- | --- | --- | --- | --- |
| Gender (%)  Male  Female | 146 (49.3)  150 (50.7) | 73 (46.2)  85 (53.8) | 0.771 | 72 (72.0)  28 (28.0) | <0.001 |
| Age (y) | 34.0  (29.0, 41.0) | 41.0  (27.8, 55.0) | 0.091 | 28.0  (23.0, 35.8) | <0.001 |
| BMI (kg/m^2^) | 22.1  (20.3, 23.7) | 22.4  (20.1, 24.5) | 0.023 | 21.6  (19.6, 24.4) | 0.600 |
| Smoking (%) | 49 (16.6) | 34 (21.5) | <0.001 | 21 (21.0) | 0.314 |
| Disease activity |  | Mayo score (%)  ≤2 0.0  3-5 11.4  6-10 58.9  11-12 29.7 |  | CDAI (%)  ≤150 52.0  150-450 48.0  >450 0.0 |  |
| Refined  grains (g/d) | 250.0  (200.0, 300.0) | 300.0  (225.0, 400.0) | <0.001 | 350.0  (300.0, 400.0) | <0.001 |
| Coarse grains (g/d) | 3.5  (0.0, 15.0) | 2.0  (0.0, 7.1) | 0.050 | 0.0  (0.0, 5.9) | 0.002 |
| Tuber (g/d) | 28.0  (14.0, 43.0) | 28.0  (14.0, 42.0) | 0.429 | 28.0  (14.0, 43.0) | 0.812 |
| Beans products (g/d) | 8.0  (3.6, 14.3) | 6.7  (2.4, 11.0) | 0.128 | 6.9  (3.5, 10.7) | 0.004 |
| Vegetables  (g/d) | 300.0  (250.0, 400.0) | 200.0  (100.0, 300.0) | <0.001 | 200.0  (100.0, 300.0) | <0.001 |
| Fruits (g/d) | 200.0  (71.4, 250.0) | 107.1  (0.0, 300.0) | 0.283 | 85.7  (22.1, 241.1) | 0.014 |
| Dairy products (g/d) | 107.1  (31.4， 242.3) | 57.1  (0.0, 200.0) | 0.017 | 100.0  (0.0, 178.6) | 0.083 |
| Eggs (g/d) | 60.0  (30.0, 60.0) | 60.0  (25.7, 60.0) | 0.245 | 60.0  (25.7, 60.00) | 0.157 |
| Meats (g/d) | 100.0  (50.0, 150.0) | 100.0  (50.0, 150.0) | 0.053 | 150.0  (72.3, 200.0) | <0.001 |
| Processed  meat (g/w) | 0.0  (0.0, 40.0) | 0.0  (0.0, 50.0) | 0.993 | 17.5  (0.0, 100.0) | 0.001 |
| Beverages  (mL/d) | 0.0  (0.0, 82.1) | 16.7  (0.0, 214.3) | 0.002 | 78.6  (0.0, 296.5) | <0.001 |
| Fried food (g/w) | 40.0  (0.0, 100.0) | 80.0  (20.0, 240.0) | 0.001 | 160.0  (65.0, 305.0) | <0.001 |
| Baked food  (g/w) | 50.0  (0.0, 150.0) | 175.0  (0.0, 400.0) | <0.001 | 200.0  (25.0, 437.5) | <0.001 |
| Fried UPF  (g/w) | 0.0  (0.0, 0.0) | 0.0  (0.0, 70.0) | <0.001 | 0.0  (0.0, 70.0) | <0.001 |
| Oil (g/d) | 30.0  (30.0, 30.0) | 30.0  (30.0, 40.0) | 0.003 | 30.0  (30.0, 40.0) | <0.001 |

Non-normally distributed continuous variables are expressed as median.

**Table S2 Food groups and food items of dietary questionnaire**

| Food groups | Food items | Weight |
| --- | --- | --- |
| Refined grains (g/d) | Rice and rice products (cooked rice, rice noodles,etc), wheat flour products (steamed buns, noodles) |  |
| Coarse grains (g/d) | Buckwheat, millet, oat, corn, etc. |  |
| Tuber (g/d) | Potatoes, sweet potatoes, taro, etc. |  |
| Legumes (g/d) | Soybeans, soy milk, tofu, fermented tofu, dried tofu skin, ready-to-eat soy products, etc. |  |
| Vegetables (g/d) | Fresh legumes (green beans, cowpeas, string beans), nightshades (eggplant, tomato, bell pepper), gourds (cucumber, zucchini), alliums (garlic chives, leeks, scallions), stem vegetables (celery, asparagus lettuce), root vegetables (radish, lotus root, yam), brassicas (cauliflower, cabbage), leafy greens (spinach, bok choy, napa cabbage), pickled vegetables |  |
| Fruits (g/d) | Citrus fruits (oranges, pomelos), pome fruits (apples, pears), stone fruits (peaches, plums, apricots), berries (strawberries, grapes), tropical fruits (mango, banana, starfruit), melons (watermelon, cantaloupe) |  |
| Dairy products (g/d) | Whole milk, low-fat/skim milk, whole milk powder, low-fat milk powder, yogurt, cheese, ice cream |  |
| Eggs (g/d) | Fresh eggs, salted duck eggs, century eggs |  |
| Meats (g/d) | Fresh/frozen/cooked pork, beef, lamb, poultry, and other meat, pork liver, pork kidney and other animal offal, fish, shrimp, crab, ect. |  |
| Processed meat (g/w) | Sausages, ham, etc. |  |
| Beverages (mL/d) | Carbonated drinks, fresh vegetable/fruit juice, flavored milk drinks, coffee, tea, etc. |  |
| Fried food (g/w) | Fried dough sticks (Youtiao), fried pancakes, fried cakes, sesame balls, ect. |  |
| Baked food (g/w) | Bread, cream cake, biscuits, cookies, other pastries |  |
| Fried UPF (g/w) | Chips, french fries, other fried puffed snacks |  |
| Oil (g/d) | Peanut oil, soybean oil, rapeseed oil, sesame oil, animal fat, butter, etc. |  |

**Table S3 Primer sequences used for Realtime-PCR.**

| Primers | Sequence（5’-3’） |
| --- | --- |
| GAPDH | F: ATGGGTGTGAACCACGAGA  R: CAGGGATGATGTTCTGGGCA |
| IL-1β | F: TCAGGCAGGCAGTATCACTC  R: AGCTCATATGGGTCCGACAG |
| IL-6 | F: CACAGAGGATACCACTCCCAACAGA  R: ACAATCAGAATTGCCATTGCACAAC |
| TNF-α | F: AGCACAGAAAGCATGATCCG  R: CTGATGAGAGGGAGGCCATT |
| PDLIM3 | F: AAGCAGCGTCCTACCAGTTGT  R: TCCTGTGGTTCAGCTTCTAAGTTG |

**Table S4.** Proteins with C terminal sequence from *Staphylococcus_Lentus* could interact with PDLIM3

| Gene | C terminal sequence | PDZ class | Description |
| --- | --- | --- | --- |
| E4T78_00965 | SISQGQDLVIP | Ⅱ | LysM domain-containing protein |
| IR128_01905 | GWAQVKQDEA | Ⅲ | Conserved virulence factor B |
| tuf | GSGVVTVIEA | Ⅱ | Elongation factor |
| E4T78_10530 | DESEEETSKV | Ⅰ | ATP-dependent Clp protease |
| glyQS | ENFLAEKIKF | Ⅱ | Glycine-tRNA ligase |
| fusA | EEIIKKNTGA | Ⅰ | Elongation factor |
| argS | LIGVSAPESM | Ⅲ | Arginine--tRNA ligase |
| gcvPB | KFELLKEEKV | Ⅲ | Probable glycine dehydrogenase |
| pruA | LQGRTTSEML | Ⅲ | L-glutamate γ-semialdehyde dehydrogenase |
| rpoY | KSEHFNVEKI | Ⅲ | DNA-directed RNA polymerase |
| E4T78_03460 | KFIERRDEFM | Ⅲ | Large polyvalent protein associated domain-containing protein |
| frr | TEKEKDILEI | Ⅱ | Ribosome-recycling factor |
| E4T78_10340 | VETEEEEVNL | Ⅱ | FMN-dependent NADPH-azoreductase |
| rpsB | KEESTETTEA | Ⅰ | Small ribosomal subunit protein |
| sufD | VIETKVTTQI | Ⅰ | Fe-S cluster assembly protein |
| E4T78_09740 | EAAFNALPEA | Ⅱ | Glycine cleavage system protein |
| ptsP | EVVELVNELA | Ⅲ | Phosphoenolpyruvate-protein phosphotransferase |
| E4T78_12475 | FVIDGGITAL | Ⅰ | Glucose 1-dehydrogenase |
| E4T78_02980 | VQVLEKHLYI | Ⅱ | GAF domain-containing protein |
| E4T78_02465 | YLKQENEFNA | Ⅱ | UPF0297 protein E4T78_02465 |
| IR128_12030 | DELEGYMFYI | Ⅱ | Rrf2 family transcriptional regulator |
| pfkA | MYELANQLSI | Ⅱ | ATP-dependent 6-phosphofructokinase |
| secG | IMLAITYFKM | Ⅱ | Protein-export membrane protein |
| rho | ESAKTGKSII | Ⅰ | Transcription termination factor Rho |
| E4T78_06275 | PKLKDEKSNI | Ⅰ | HTH-type transcriptional regulator Hpr |
| rplO | EGKGGTHEVI | Ⅲ | Large ribosomal subunit protein uL15 |
| rpsJ | PSGVDIEIKL | Ⅱ | Small ribosomal subunit protein uS10 |
| yhbY | SKENKKIELP | Ⅲ | Ribosome assembly RNA-binding protein YhbY |
| murA | EGKLKDKARV | Ⅱ | UDP-N-acetylglucosamine 1-carboxyvinyltransferase |
| rnj | PMIIPIISEI | Ⅰ | Ribonuclease |
| rplN | KIVSLAPEVL | Ⅲ | Large ribosomal subunit protein uL14 |
| E4T78_07590 | YSHIEENWIF | Ⅱ | UPF0349 protein E4T78_07590 |
| E4T78_03340 | DIKASGLSIL | Ⅰ | Transaldolase |
| E4T78_11925 | EVFLKDGVVL | Ⅱ | Metal ABC transporter ATP-binding protein |
| cspA | GPQAANVVKL | Ⅱ | Cold shock protein CspA |
| E4T78_13140 | LLDEQSEDDA | Ⅲ | HTH-type transcriptional regulator Hpr |
| E4T78_14150 | YLNNYFDLNM | Ⅱ | Cof-type HAD-IIB family hydrolase |
| E4T78_04995 | QLRNAIEEVL | Ⅲ | Glutathione peroxidase |
| E4T78_02455 | EIVNTEMDDI | Ⅲ | UPF0473 protein E4T78_02455 |
| rplU | TKLTIEKINA | Ⅱ | Large ribosomal subunit protein bL21 |
| clpX | NLLNDEKTSA | Ⅰ | ATP-dependent Clp protease ATP-binding subunit |
| E4T78_01095 | ALFEEHCDEI | Ⅲ | BrxA/BrxB family bacilliredoxin |
| rpsA | FGDKLKNFKL | Ⅱ | 30S ribosomal protein S1 |
| E4T78_01105 | EQKMRTLAEF | Ⅱ | Alpha-ketoacid dehydrogenase subunit beta |
| E4T78_09975 | RALTGDSAFL | Ⅱ | Glutaryl-CoA dehydrogenase |
| ssb | IDISDDDLPF | Ⅱ | Single-stranded DNA-binding protein |
| E4T78_01110 | VYDQGGESNA | Ⅰ | 2-oxoisovalerate dehydrogenase subunit alpha |
| E4T78_07800 | KEDEYRQTWL | Ⅰ | Arsenate reductase family protein |
| E4T78_07840 | IDVKLNVEAV | Ⅲ | Organic hydroperoxide resistance protein |
| E4T78_06325 | AIFDAGYEVI | Ⅲ | Heavy-metal-associated domain-containing protein |
| rplA | GIKVDTSNFKL | Ⅱ | Large ribosomal subunit protein uL1 |
| rplQ | GAESVIIELV | Ⅲ | Large ribosomal subunit protein bL17 |
| atpD | ENAKNMGVEV | Ⅱ | ATP synthase subunit beta |
| E4T78_10920 | KQLIKDMEEL | Ⅲ | Winged helix-turn-helix transcriptional regulator |
| serS | FMGGQTVIEL | Ⅱ | Serine--tRNA ligase |
| E4T78_10150 | ILAVFEEVEL | Ⅱ | Mannitol-specific phosphotransferase enzyme IIA component |
| E4T78_03570 | KLHDAFTNA | Ⅰ | UPF0358 protein E4T78_03570 |
| IR128_01190 | IGNGDSIFDF | Ⅱ | DUF5327 family protein |
| rbsK | LSEIEGEWHV | Ⅱ | Ribokinase |
| E4T78_04410 | VRVINDDEEA | Ⅲ | Alkaline shock protein 23 |
| pgi | DLKKDLEDRL | Ⅲ | Glucose-6-phosphate isomerase |
| E4T78_13665 | TENAERLFKL | Ⅱ | TatD family deoxyribonuclease |
| E4T78_14000 | EHPSVKEFNI | Ⅱ | Sugar phosphate isomerase/epimerase |
| prfC | FPEIKLYSLL | Ⅰ | Peptide chain release factor 3 |
| E4T78_13150 | EIAKAIQSQF | Ⅰ | HIT family protein |
| mcsB | KRATFLRENI | Ⅲ | Protein-arginine kinase |
| E4T78_08405 | PVFRDGNWAF | Ⅱ | Aminopeptidase |
| E4T78_12005 | IIVIMAHIFF | Ⅱ | YjiH family protein |
| E4T78_09390 | EVGASQWVTA | Ⅱ | Transglycosylase |
| nusG | EVEFDQIEKL | Ⅲ | Transcription termination/antitermination protein NusG |
| rplR | DAARENGLQF | Ⅱ | Large ribosomal subunit protein uL18 |
| E4T78_12735 | EQDPAEYEEF | Ⅲ | Aldo/keto reductase |
| E4T78_03480 | VAQAKETLEF | Ⅱ | Alpha-ketoacid dehydrogenase subunit beta |
| E4T78_10825 | FGPKMSITNV | Ⅰ | nitric oxide dioxygenase |
| dapB | TYNNLNEERI | Ⅲ | 4-hydroxy-tetrahydrodipicolinate reductase |
| E4T78_10550 | LSATIAGFFL | Ⅱ | NupC/NupG family nucleoside CNT transporter |
| rpoE | DDDEEEEEEL | Ⅲ | Probable DNA-directed RNA polymerase subunit delta |
| hemL | KAADIALSQL | Ⅰ | Glutamate-1-semialdehyde 2,1-aminomutase |
| murT | VLNRAFKEEV | Ⅲ | Lipid II isoglutaminyl synthase (glutamine-hydrolyzing) subunit MurT |
| E4T78_05105 | VQQVYHNVEL | Ⅱ | Probable transcriptional regulatory protein E4T78_05105 |
| E4T78_01130 | HSICCALTLV | Ⅰ | Phosphate acetyltransferase |
| E4T78_02890 | ELARDIKSEI | Ⅰ | Bifunctional oligoribonuclease/PAP phosphatase NrnA |
| E4T78_04910 | EIASNMKELI | Ⅲ | 2-oxoacid:acceptor oxidoreductase subunit alpha |
| E4T78_01200 | TKSTKDLIIL | Ⅱ | Aminopeptidase P family protein |
| murA | LGADIWTEEV | Ⅲ | UDP-N-acetylglucosamine 1-carboxyvinyltransferase |
| hemC | IIRKLNEEEA | Ⅲ | Porphobilinogen deaminase |
| trmFO | KHLDSFKVML | Ⅱ | Methylenetetrahydrofolate--tRNA-(uracil-5-)-methyltransferase TrmFO |
| asnS | FPRLLNRLYP | Ⅱ | Asparagine--tRNA ligase |
| tkt | ENVLNTVLSF | Ⅱ | Transketolase |
| IR128_12865 | LETKVASILL | Ⅱ | Aldehyde dehydrogenase |
| IR128_04015 | NEKLENIWWA | Ⅱ | Sugar phosphate isomerase/epimerase |
| IR128_07770 | SGEDVVIWTP | Ⅱ | Uncharacterized N-acetyltransferase IR128_07770 |
| ureA | KLITVHHPIV | Ⅱ | Urease subunit gamma |
| ureC | LPLTQRYFLF | Ⅱ | Urease subunit alpha |
| IR128_08760 | PKRVERVTF | Ⅱ | ABC transporter ATP-binding protein |
| iolA | VTARHGDPQF | Ⅱ | Malonate-semialdehyde dehydrogenase |
| IR128_08560 | KKEDIIDLML | Ⅱ | lipoate--protein ligase |
| IR128_11705 | AQIKQIKEVL | Ⅲ | tRNA (Adenine(22)-N(1))-methyltransferase TrmK |
| gyrB | ENAEYATLDI | Ⅱ | DNA gyrase subunit B |
| thiD | KIEVYTEEV | Ⅲ | Bifunctional hydroxymethylpyrimidine kinase/phosphomethylpyrimidine kinase |
| IR128_05435 | NDDTVKDIMW | Ⅱ | M42 family metallopeptidase |
| phnW | NHYMEERAYA | Ⅱ | 2-aminoethylphosphonate--pyruvate transaminase |
| IR128_07805 | KELEKQSLAL | Ⅱ | Phosphoesterase |
| kynU | QYSNKRGLVV | Ⅱ | Kynureninase |
| pruA | LEQKILSEQF | Ⅲ | 1-pyrroline-5-carboxylate dehydrogenase |
| IR128_05830 | SFETKNIITL | Ⅱ | Gfo/Idh/MocA family oxidoreductase |
| gpsA | MERDKKSENI | Ⅲ | Glycerol-3-phosphate dehydrogenase [NAD(P)+] |
| IR128_11595 | GTYVIDKENA | Ⅲ | Glucokinase |
| IR128_01295 | DIINLLNELV | Ⅲ | HD domain-containing protein |
| ychF | GDVVHFRFNV | Ⅱ | Ribosome-binding ATPase YchF |
| ccsB | LIVVGLHSYA | Ⅰ | C-type cytochrome biogenesis protein CcsB |
| yqeH | VEVTSRKSIL | Ⅰ | Ribosome biogenesis GTPase YqeH |
| IR128_12810 | LELTLKSEGL | Ⅲ | Uncharacterized protein |
| IR128_05615 | TEVKFVWVKL | Ⅱ | Aldehyde dehydrogenase family protein |
| IR128_02240 | LVKAARLSTF | Ⅰ | C40 family peptidase |
| IR128_14550 | EITQDLNEEI | Ⅲ | DUF2529 family protein |
| IR128_03920 | SYCLIAEVLL | Ⅱ | LLM class flavin-dependent oxidoreductase |
| IR128_00670 | ATLNIDQSEF | Ⅰ | M3 family oligoendopeptidase |
| IR128_08275 | FGPKMSITNV | Ⅰ | nitric oxide dioxygenase |
| IR128_01505 | DGGFMAYSGV | Ⅰ | SDR family oxidoreductase |
| uvrA | LKPILERDRA | Ⅲ | UvrABC system protein A |
| murD | ESFQKHLPSF | Ⅱ | UDP-N-acetylmuramoylalanine--D-glutamate ligase |
| IR128_10495 | EAVLAAKEIL | Ⅲ | Alpha/beta hydrolase |
| fabH | YSSSLIKWGI | Ⅱ | Beta-ketoacyl-[acyl-carrier-protein] synthase III |
| IR128_11700 | NINTDPYEYM | Ⅲ | GTP cyclohydrolase 1 type 2 homolog |
| thiO | RFKAKRSVEL | Ⅱ | Glycine oxidase ThiO |
| IR128_03955 | RALTGDSAFL | Ⅱ | Acyl-CoA dehydrogenase family protein |
| IR128_12870 | IMDELEKFNP | Ⅱ | NERD domain-containing protein |
| IR128_02290 | EEIESNLEPI | Ⅲ | VOC family protein |
| IR128_04720 | ARGFRSMLRV | Ⅱ | DUF4256 domain-containing protein |
| dhaL | FVLNALSEDV | Ⅲ | Dihydroxyacetone kinase subunit L |
| dgoD | RHKDGSIAEW | Ⅱ | Galactonate dehydratase |
| IR128_02460 | KGFFQRLFNI | Ⅱ | DNA-binding protein |
| aroC | KQHRDWNLNF | Ⅱ | Chorismate synthase |
| IR128_09680 | QALRLLKSNL | Ⅰ | Glycerate kinase |
| IR128_13990 | HQSYESNLNI | Ⅱ | Molybdopterin molybdenumtransferase |
| IR128_12180 | FISEVRKLLV | Ⅱ | Bifunctional folylpolyglutamate synthase/dihydrofolate synthase |
| hslV | TNHEIRVEEI | Ⅲ | ATP-dependent protease subunit HslV |
| bshC | YTFEHITIEV | Ⅱ | Putative cysteine ligase BshC |
| IR128_08755 | YNKTFNLSEA | Ⅰ | ATP-binding cassette domain-containing protein |
| IR128_12390 | ILKPGEHVEL | Ⅱ | UPF0173 metal-dependent hydrolase IR128_12390 |
| ffh | NPFQGMNLPF | Ⅱ | Signal recognition particle protein |
| pyrE | KTLKEWKDTL | Ⅲ | Orotate phosphoribosyltransferase |
| IR128_14605 | GHRFKGEDEL | Ⅲ | Threonylcarbamoyl-AMP synthase |
